# N-Orbit: towards a universal model and metric for comparing tissue microenvironments

**DOI:** 10.1038/s41467-026-73561-8

**Published:** 2026-05-22

**Authors:** Barbara Xiong, Yuxuan Hu, Kai Tan

**Affiliations:** 1 Graduate Group in Genomics and Computational Biology, Perelman School of Medicine, University of Pennsylvania, Philadelphia, PA USA; 2 Medical Scientist Training Program, Perelman School of Medicine, University of Pennsylvania, Philadelphia, PA USA; 3 School of Computer Science and Technology, Xidian University, Xi’an, Shaanxi China; 4 Department of Pediatrics, Perelman School of Medicine, University of Pennsylvania, Philadelphia, PA USA; 5 Division of Oncology and Center for Childhood Cancer Research, Children’s Hospital of Philadelphia, Philadelphia, PA USA; 6 Center for Single Cell Biology, Children’s Hospital of Philadelphia, Philadelphia, PA USA

**Keywords:** Computational biology and bioinformatics, Computational models, Gene expression, Transcriptomics, Data processing

## Abstract

Spatial omics technologies facilitate comprehensive exploration of tissue microenvironments across development and disease. Yet a theoretical framework for modeling and comparing tissue architecture in diverse biological contexts remains lacking. We introduce N-Orbit, a mathematical model that captures both cell-type composition and spatial relationships within tissue cellular neighborhoods, encoding them as vectors for efficient distance calculations. While not a neighborhood detection method itself, N-Orbit enhances insights gleaned from neighborhoods generated by the plethora of recently developed methods. We benchmark the N-Orbit-based neighborhood distance metric on spatial omics datasets that include ground-truth neighborhoods and clinical outcomes. We demonstrate that N-Orbit outperforms cell-type-enrichment-based metrics in discriminating among neighborhood types, predicting clinical variables, and identifying homologous structures across species. Additionally, N-Orbit enhances model interpretability by tracing neighborhoods back to enriched spatial motifs. N-Orbit holds significant potential for deepening our understanding of how tissue microenvironments remodel during development, disease, and evolution.

## Abstract (web summary)

Spatial omics technologies facilitate comprehensive exploration of tissue microenvironments across development and disease. Here, the authors introduce N-Orbit, a theoretical framework for modeling and comparing tissue architecture in diverse biological contexts using a distance-based approach.

## Introduction

The rapid advancement of spatial omics technologies has revolutionized the study of tissue microenvironments (TMEs). Imaging-based technologies, such as multiplex error-robust fluorescence in situ hybridization (MERFISH) for RNAs and co-detection-by-InDEXing (CODEX) for proteins, use multiplexed in situ hybridization of fluorescent probes^1,2^. Sequencing-based technologies, including STARmap and Stereo-Seq, employ microarrays of spatially barcoded probes to measure RNA expression with single-cell spatial resolution^3,4^. By leveraging spatial transcriptomic and proteomic data, researchers can map the diverse landscape of intermingling cell types, uncovering spatial heterogeneity and cell–cell interactions.

Tissue cellular neighborhoods (TCNs) are fundamental organizational units of tissue architecture^5^. They are spatially contiguous tissue regions with consistent cell type compositions that perform coordinated function(s). The TCN’s phenotype reflects the collective behavior of its constituent cells: how they communicate and organize to perform specialized functions. Identifying TCNs is crucial for understanding tissue development, homeostasis, and disease mechanisms. Several computational tools have been developed to detect TCNs from spatial omics data, including our recently developed method, CytoCommunity^6–11^. Partitioning spatial omics maps into TCNs enables the identification of localized patterns that may otherwise be diluted and overlooked in bulk analyses. This is particularly important in spatially heterogeneous contexts.

Analyzing TCNs over time may capture dynamic processes crucial to understanding disease progression, such as cellular proliferation, migration, and reorganization, during which TCNs may undergo significant alterations. Monitoring these changes provides insights into disease pathophysiology and serves as a marker of treatment efficacy and clinical outcomes.

Comparing TCNs across species can also provide critical insights into the conserved roles and behaviors of cellular organizations over evolutionary time. While individual cell types may vary between species in structure or function, analysis of conserved TCNs allows us to better understand the evolutionary selective pressures on cellular communication and the conservation of functional tissue architecture.

While the problem of TCN detection itself has been thoroughly explored, a fundamental open question is how to quantitatively characterize changes in the resulting TCNs across various conditions, a challenge that is of similar significance and broad applicability to differential gene expression analysis. Developing quantitative methods to compare patient samples is particularly difficult because these samples are often derived from physically distinct tissues. The absence of physical continuity and the sparsity of time points complicate tracing spatial rearrangements. Consequently, previous attempts have primarily analyzed TCNs only at the level of cell-type enrichment. There has been a lack of principled mathematical models for describing TCN structure. It is also infeasible to directly align individual cells from one sample to another to track spatial changes. Furthermore, no methods exist to compare TCNs across species, a challenge analogous to the multiple sequence alignment problem in homology detection^12^. Alignment-based methods are limited by their computational complexity and sensitivity to highly divergent sequences. However, *k-mer*-based methods, which account for word frequencies, have proven to be an effective heuristic technique with substantially greater robustness and scalability^13^. We draw inspiration from this approach and adapt it to the concept of TCNs.

Here, we introduce the N-Orbit, an interpretable mathematical model for the building block of TCNs. The N-Orbit model is applicable to single-cell-resolution spatial omics data, in which each cell is assigned a type and spatial coordinates. By applying the N-Orbit model, we define a distance metric to quantify differences between pairs of TCNs in an alignment-free manner. Using the TCN distance metric, we can construct an overall distance matrix for the entire dataset, enabling projection of TCNs onto a common low-dimensional embedding for visualization and global comparison. We also formulate an N-Orbit enrichment test that enables tracing TCN changes between conditions to the relevant N-Orbit structures, thereby enhancing interpretability.

We systematically evaluated the N-Orbit model and its associated distance metric through several key assessments. First, we tested its ability to distinguish analogous TCNs from unrelated ones across datasets spanning diverse modalities and scales. Second, we examined its utility in capturing biologically and clinically relevant information. Third, we assessed its ability to identify homologous TCNs across aging and species. Overall, we demonstrated the potential of this framework to augment insights gained from the myriad of recently developed TCN detection methods, as well as its broad applicability across diverse TME contexts.

## Results

### Overview of the N-Orbit framework

**Fig. 1:**
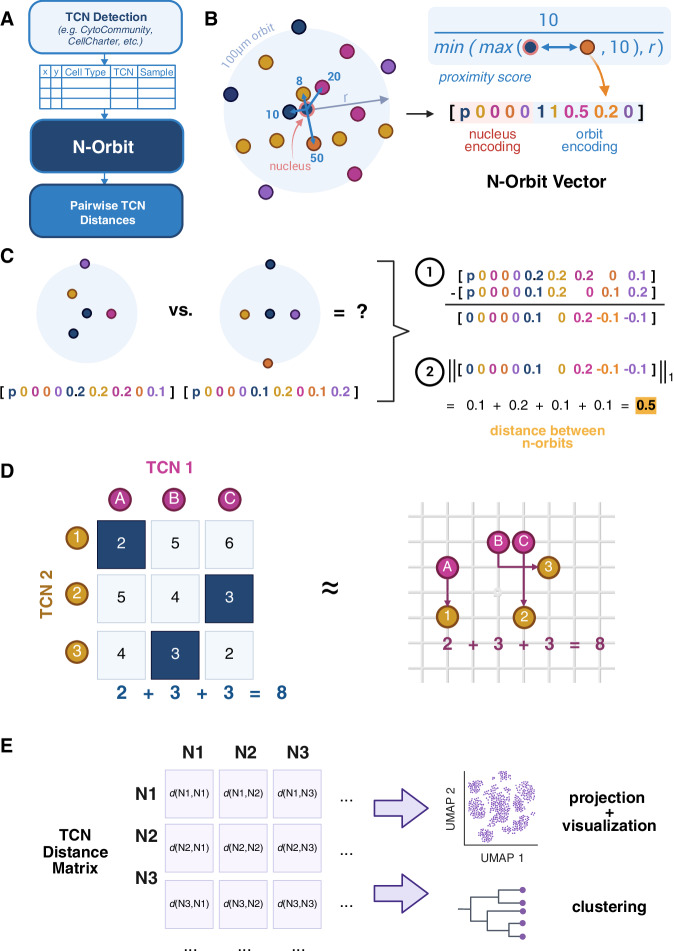
Schematic of the N-Orbit framework. **A** Before performing N-Orbit analysis, tissue cellular neighborhoods (TCNs) are assigned using a separate detection method, such as CytoCommunity or CellCharter. From these pre-specified TCNs, N-Orbit computes pairwise distances between them based on the spatial proximity of constituent cell types. **B** Derivation of an N-Orbit from the *r*-radius area surrounding the cell of interest, along with the corresponding vector encoding. The vector encoding comprises nucleus and orbit components, where each index corresponds to a possible cell type. The nucleus penalty change *p* is applied at the nucleus encoding index corresponding to the center cell type, with all other elements set to zero. The orbit encoding contains proximity scores for each possible cell type, based on the shortest distance to a cell of that type. Distances are clipped between 10 µm and *r* µm, or 100 µm in this example. **C** Example calculation of distance between two N-Orbit structures as the Manhattan distance between their vector representations. **D** Example calculation of N-Orbit-based distance between two TCNs as the minimum total distance between the N-Orbits in TCN 1 and TCN 2, determined by a cost matrix optimization. **E** Compilation of the N-Orbit-based TCN distance matrix, which serves as an input for projection, visualization, and clustering analysis. Images were created in BioRender. Xiong, B. (2026) https://BioRender.com/f57e947. Requirements of User: (1) All Completed Graphics to be published in any publication (journals, textbooks, websites, etc.) must be accompanied by the following citation either as a caption, footnote or reference for each figure that includes a Completed Graphic: “Created in BioRender. Xiong, B. (2026) https://BioRender.com/f57e947 “. (2) All terms of the License Terms, including all Prohibited Uses are fully complied with. E.g., for Academic License Users, no commercial uses (beyond publication in journals, textbooks or websites) are permitted without obtaining or switching to a BioRender Industry Plan. (3) A reader may request that the user allow their figure to be a public template for readers to view, copy, and modify the figure. It is up to the user to determine what level of access to grant.

The N-Orbit framework was designed to accept TCNs identified by other methods as input (Fig. 1A). We describe the *N-Orbit* of a cell as a snapshot of its local surroundings, particularly its shortest physical distance to all possible cell types (Fig. 1B). Each N-Orbit is representable as a vector, encoding both the identity of the center cell type (the *nucleus*) and its reachability (i.e., inverse distance) to all cell types. With this vector formulation, the *edit distance between two N-Orbits* can be computed algebraically by their Manhattan distance (Fig. 1C). The *edit distance between two TCNs* is then defined as the minimum total distance between the set of N-Orbits representing each TCN (Fig. 1D). These pairwise TCN distances can be compiled into an overall *TCN distance matrix* (Fig. 1E). Sample-level distances are calculated analogously.

We compared the performance of our N-Orbit-based TCN distance metric with a naïve approach based on cell-type enrichment (CTE). We assessed their ability to discriminate between pairs of TCNs of varying relatedness with the area under the receiver operating characteristic (AUROC) of distance-thresholded classifiers. Similarly, we compared N-Orbit with graph-based TCN detection methods that generate cell-level embeddings as an intermediate step, using embedding distance to distinguish cell pairs. Performance metrics for benchmarking, including p-values, are provided in Supplementary Tables 2 and 3 for reference. Hyperparameters used for N-Orbit are also provided in Supplementary Table 4. The source code for the N-Orbit software has been deposited on GitHub^14^ and can be downloaded from https://github.com/tanlabcode/N-Orbit.

### Evaluation of the N-orbit model using synthetic TCNs

**Fig. 2:**
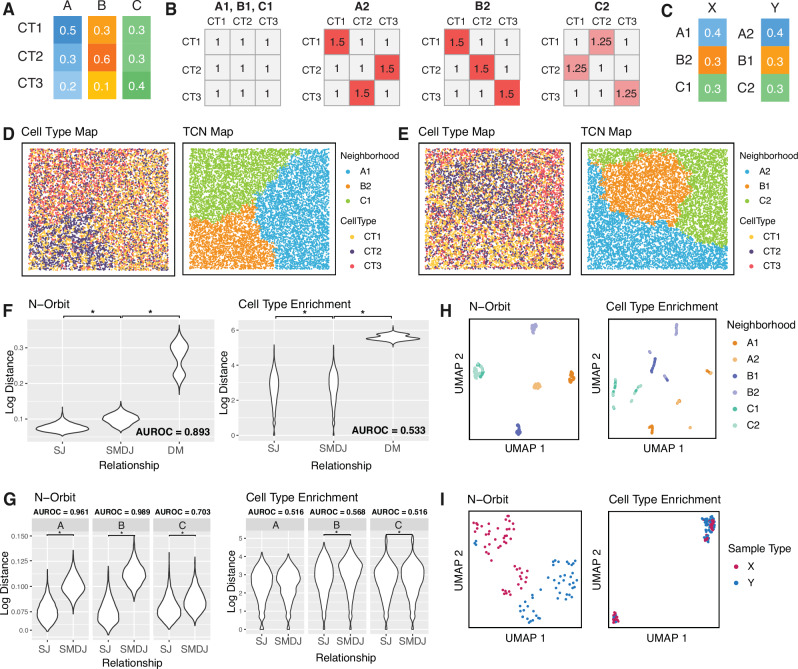
Performance evaluation of the N-Orbit model to distinguish synthetic tissue cellular neighborhoods (TCNs) of similar marginal but different joint distributions. **A** Cell type (CT) compositions for each TCN classes A–C. **B** Cell type interactions (edge potentials) for each TCN. **C** Proportions of each TCN within sample types X and Y. **D** Example cell type and TCN maps for Sample Type X. **E** Example cell type and TCN maps for Sample Type Y. **F** Violin plots of log (scaled) N-Orbit and Cell Type Enrichment (CTE)-based TCN distances for TCN pairs with same joint (SJ), same marginal but different joint (SMDJ), and different marginal (DM) distributions. Asterisks indicate statistical significance by one-sided *t*-test (N-Orbit: SJ vs. SMDJ <1e − 324, SMDJ vs. DM <1e − 324. CTE: SJ vs. SMDJ = 3.34e − 9, SMDJ vs. DM <1e − 324). The area under the receiver operating characteristic curve (AUROC) is provided for the SJ vs. SMDJ comparison. **G** Violin plots of log (scaled) N-Orbit and CTE-based TCN distances for SJ and SMDJ TCN pairs within each TCN class. Asterisks indicate statistical significance by one-sided *t*-test (N-Orbit: A < 1e − 324, B < 1e − 324, C = 3.16e − 124. CTE: A = 0.17, B = 7.6e − 13, C = 0.05). AUROC is provided for the SJ vs. SMDJ comparison. **H** UMAPs of N-Orbit and CTE TCN distance matrices, color-coded by TCN. Each point represents one TCN. **I** UMAPs of N-Orbit and CTE sample distance matrices, color-coded by sample type. Each point represents one sample. Source data are provided as a Source Data file.

We first simulated 100 synthetic samples to validate N-Orbit’s ability to capture spatial relationships beyond cell type composition (Fig. 2A–E). We designed synthetic TCNs comprising three cell types (CT1, CT2, CT3) with similar marginal distributions (reflecting the overall cell-type composition) but different pairwise joint distributions (representing cell-type interactions). Three broad classes of TCNs (A, B, and C) were generated, each with varying marginal distributions. Within each class, one subtype (A1, B1, C1) had cell types distributed independently, whereas the other (A2, B2, C2) exhibited a mixture of monotypic and heterotypic cell-type interactions. These were grouped into two sample types (X, Y), each containing one TCN type from each broad class. These sample types were designed to model clinical variability influencing TCN structure in real patient datasets.

The N-Orbit distance metric significantly outperformed CTE distance in discriminating between TCN pairs of the same joint distribution (SJ) from pairs of the same marginal but different joint distributions (SMDJ) (AUROC: N-Orbit = 0.893, CTE = 0.53; DeLong test *p*-value < 1e − 324) (Fig. 2F, G). N-Orbit also demonstrated better performance within each broad TCN class (*p*-value: A < 1e − 324, B < 1e − 324, C = 3.05e − 64). Uniform Manifold Approximation and Projections (UMAPs)^15^ based on TCN distance matrices revealed clear separation of TCN subtypes within the same broad class when using N-Orbit but not CTE (Fig. 2H). Additionally, sample-level UMAPs exhibited near-perfect separation between sample types X and Y using N-Orbit, while CTE resulted in intermixing (Fig. 2I). Overall, these results underscore N-Orbit’s superiority in capturing subtle nuances of cell-type interactions compared to the naïve CTE distance metric.

We simulated another 100-sample dataset containing eight cell types and five TCN types, each with the same cell-type distribution but varying pairwise joint distributions (Supplementary Fig. 1A–D). TCN types were grouped into two sample types, X (comprising N1, N2, and N4) and Y (containing N1, N3, and N5). Again, N-Orbit significantly outperformed CTE in distinguishing TCN pairs of the same versus different types (AUROC: N-Orbit = 0.77, CTE = 0.56; *p*-value < 1e − 324) (Supplementary Fig. 1E). Principal component analysis (PCA) plots at both the TCN and sample levels also showed better separation of TCN and sample types with N-Orbit than with CTE (Supplementary Fig. 1F, G).

### Evaluation of the N-Orbit model on manually annotated TCNs

#### Mouse spleen TCNs

**Fig. 3:**
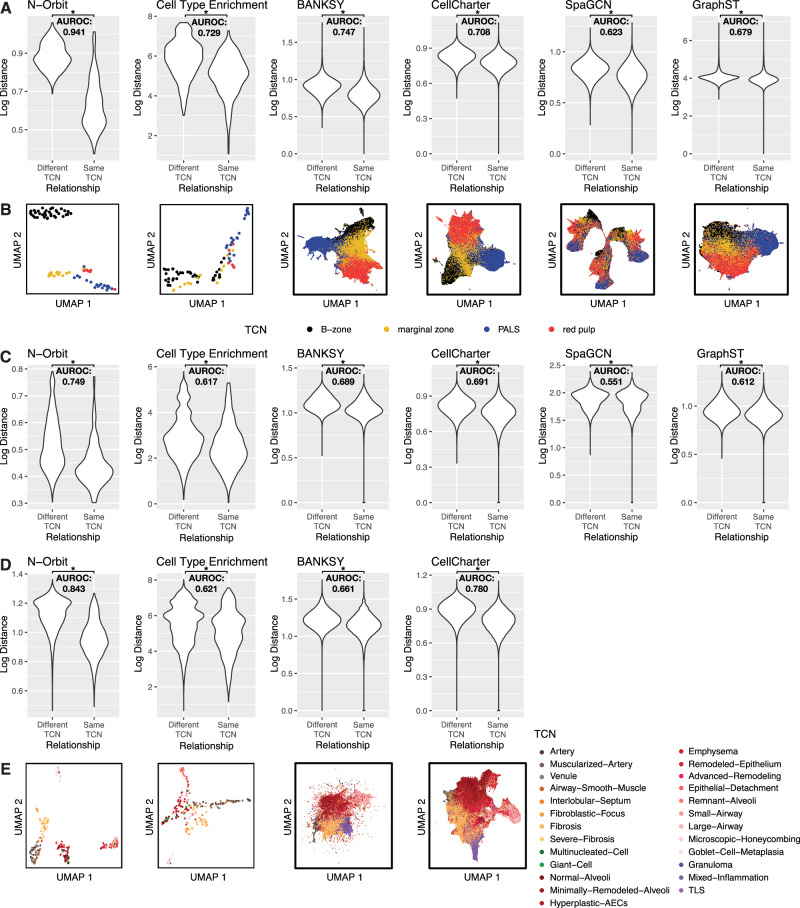
Performance evaluation of the N-Orbit model to distinguish ground truth structures. **A** Violin plots of log (scaled) N-Orbit, cell-type enrichment (CTE), and graph embedding-based distances from similar vs. dissimilar mouse spleen tissue cellular neighborhoods (TCNs) of the same CODEX sample. For N-Orbit and CTE, distances are between pairs of TCN instances. For BANKSY, CellCharter, SpaGCN, and GraphST, distances are between pairs of cells. AUROCs are shown. Asterisks indicate statistical significance by one-sided *t*-test (N-Orbit: *p* = 3.42e − 171, CTE: *p* = 2.4e − 24, BANKSY: *p* < 1e − 324, CellCharter: *p* < 1e − 324, SpaGCN: *p* = 1.93e − 124, GraphST: *p* < 1e − 324). **B** UMAPs of N-Orbit, CTE, and embedding-based distance matrices for the spleen CODEX dataset, color-coded by TCN type. For N-Orbit and CTE, each point is a TCN instance. For all others, each point is a cell. **C** Violin plots of log (scaled) N-Orbit, CTE, and graph embedding-based distances from similar vs. dissimilar MERFISH mouse hypothalamus TCNs. For N-Orbit and CTE, distances are between pairs of TCN instances. For BANKSY, CellCharter, SpaGCN, and GraphST, distances are between pairs of cells. AUROCs are provided. Asterisks indicate statistical significance by one-sided *t*-test (N-Orbit: *p* = 8.11e − 34, CTE: *p* = 2.30e − 6, BANKSY: *p* < 1e − 324, CellCharter: *p* < 1e − 324, SpaGCN: *p* < 1e − 324, GraphST: *p* < 1e − 324). **D** Violin plots of log (scaled) N-Orbit, CTE, and graph embedding-based distances from similar vs. dissimilar Xenium human pulmonary fibrosis TCNs. For N-Orbit and CTE, distances are between pairs of TCN instances. For BANKSY and CellCharter, distances are between pairs of cells. AUROCs are provided. Asterisks indicate statistical significance by one-sided *t*-test (N-Orbit: *p* < 1e − 324, CTE: *p* = 1.75e − 46, BANKSY: *p* < 1e − 324, CellCharter: *p* < 1e − 324). **E** UMAPs of N-Orbit, CTE, and embedding-based distance matrices for the Xenium pulmonary fibrosis dataset, color-coded by TCN type. For N-Orbit and CTE, each point is a TCN instance. For BANKSY and CellCharter, each point is a cell. Source data are provided as a Source Data file.

Goltsev et al. provide a CODEX dataset of three healthy mouse spleens, manually annotated into four anatomical TCN types: red pulp, B-cell zone (B-zone), marginal zone, and peri-arterial lymphoid sheath (PALS) (Supplementary Table 1, Supplementary Fig. 2A)^16^. We used these manually annotated TCN types as ground-truth labels to assess whether our N-Orbit distance metric could accurately discriminate between instance pairs of the same versus different types within the same CODEX sample (Fig. 3A). For this task, N-Orbit significantly outperformed CTE (AUROC: N-Orbit = 0.94, CTE = 0.73; *p*-value = 1.86e − 25). It also outperformed cell-level embedding distances for graph-based TCN detection methods: BANKSY^8^ (0.75), CellCharter^9^ (0.71), SpaGCN^10^ (0.62), and GraphST^11^ (0.68). Additionally, projecting each TCN distance matrix with UMAP revealed clearer separation between TCN types with N-Orbit than with CTE (Fig. 3B).

#### Mouse hypothalamus TCNs

We performed a similar analysis on manually annotated MERFISH mouse hypothalamic nuclei from Moffitt et al.^17^. This dataset comprises 17 TCN types across five mouse hypothalamus slices (Supplementary Table 1, Supplementary Fig. 2B). We evaluated the ability of the distance metrics of N-Orbit and other methods to distinguish TCN instances derived from the same versus different hypothalamic nuclei (Fig. 3C). N-Orbit significantly outperformed CTE in discerning between similar and dissimilar pairs of TCN instances (AUROC: N-Orbit = 0.75, CTE = 0.62; *p*-value = 1.72–9). Additionally, N-Orbit outperformed cell-level embedding distances from BANKSY (0.69), CellCharter (0.69), SpaGCN (0.55), and GraphST (0.61).

#### Human pulmonary fibrosis TCNs

We also benchmarked N-Orbit on healthy and fibrotic lung TCNs from human samples reported by Vannan et al.^18^. This dataset includes 45 Xenium samples with 25 unique TCN annotations (246 total TCNs) for manually assigned histopathological structures, grouped into five categories: epithelial, vascular, immune, mesenchymal/interstitial, and general (Supplementary Table 1, Supplementary Fig. 2C). N-Orbit greatly outperformed CTE in distinguishing TCN pairs from either group (AUROC: N-Orbit = 0.84, CTE = 0.62; *p*-value = 4.88–161) (Fig. 3D). N-Orbit also demonstrated better performance than cell-level embedding distances from BANKSY (0.66) and CellCharter (0.78). SpaGCN and GraphST could not process this dataset due to memory restrictions. UMAPs demonstrated better separation among the five categories of histopathological structures with N-Orbit than with CTE (Fig. 3E).

We evaluated the performance of N-Orbit across a range of values for three key parameters, including nucleus penalty, search radius, and vector sample size. We found that N-Orbit was robust to parameter changes (Supplementary Fig. 3A–D). N-Orbit also had fast run times on the three relatively large datasets (Supplementary Fig. 3E, F). Furthermore, we compared N-Orbit to a density-based distance metric rather than a nearest-proximity-based one across all three datasets. N-Orbit performed substantially better on the spleen and pulmonary fibrosis datasets across all tested search radii (Supplementary Fig. 3G). N-Orbit also demonstrated greater stability with respect to the radius parameter and maintained higher accuracy in larger fields. Finally, N-Orbit showed stable performance on TCNs generated by BANSKY and CellCharter across various hyperparameters for those methods (Supplementary Fig. 3H, I). Stability was most notable in the spleen dataset, which had higher adjusted mutual information (AMI) scores with ground-truth TCNs. For context, AMIs on the spleen dataset ranged from 0.5 to 0.6, compared to from 0.2 (for BANKSY) to 0.35 (for CellCharter) on the hypothalamus dataset.

### Therapy response prediction using N-Orbit distances

#### Triple-negative breast cancer

**Fig. 4:**
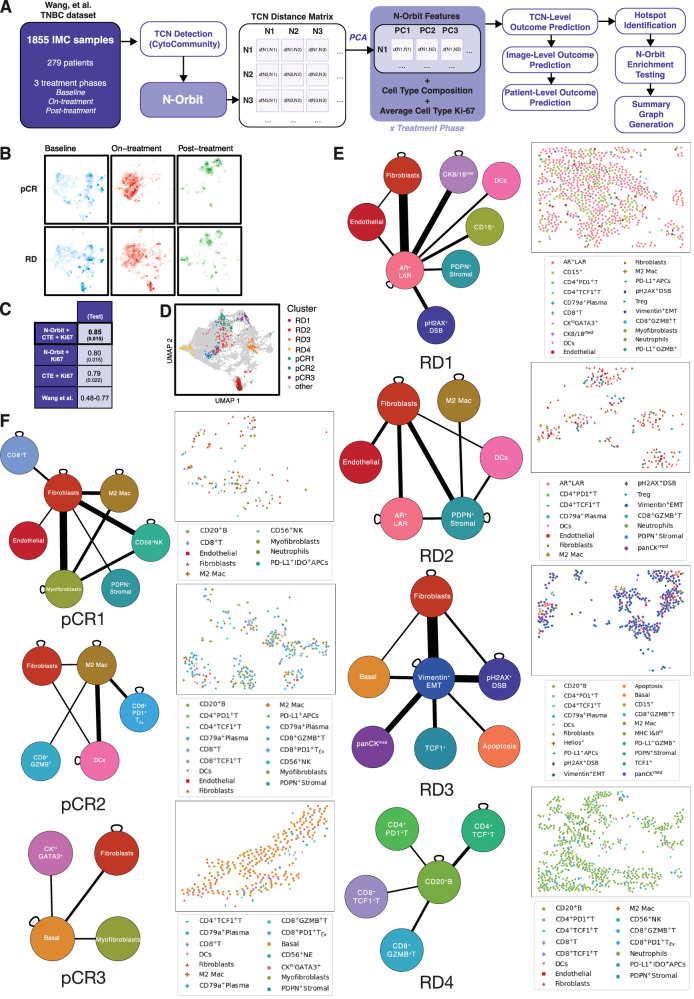
Application of N-Orbit distance to predict treatment response in triple-negative breast cancer (TNBC) patients treated with chemotherapy with or without immunotherapy. **A** Methods schematic for patient outcome prediction using N-Orbit-derived features and the generation of summary graphs. **B** UMAP density plots for the N-Orbit-based tissue cellular neighborhood (TCN) distance matrix, faceted by treatment phases (Baseline, On-treatment, Post-treatment) as columns and treatment outcomes (Residual Disease [RD], pathological complete response [pCR]) as rows. **C** Performance statistics for treatment outcome predictions from N-Orbit and CTE-based classifiers, in addition to the model from Wang et al. Standard deviations are shown in parentheses. **D** RD and pCR TCN hotspots (RD1–4, pCR1–3) plotted on the UMAP of the TCN distance matrix. TCNs outside hotspots are plotted in grey for reference. **E** Summary graphs of RD TCN hotspots, derived from their enriched N-Orbits, with corresponding example cell type maps. Edge weights indicate the relative recurrence of cell type co-memberships among enriched N-Orbits. Self-edges indicate monotypic nucleus–orbit relationships of that cell type. **F** Summary graphs of pCR TCN hotspots, derived from their enriched N-Orbits, with corresponding example cell type maps. Edge weights indicate the relative recurrence of cell type co-memberships among enriched N-Orbits. Self-edges indicate monotypic nucleus-orbit relationships of that cell type. Source data are provided as a Source Data file.

We evaluated the utility of N-Orbit in predicting treatment outcomes on two cancer datasets. The first dataset, from Wang et al., comprises 1855 imaging mass cytometry (IMC) tissue sections from 279 TNBC patients treated with chemotherapy with (C + I) or without (C) anti-PD-L1 immunotherapy, at three time points (Baseline, On-Treatment, Post-Treatment)^19^. Treatment outcomes for each patient were classified as either pathological complete response (pCR, 150 patients) or residual disease (RD, 129 patients) (Supplementary Table 1, Supplementary Fig. 2D). We detected TCNs using CytoCommunity and used N-Orbit to generate a dataset-wide TCN distance matrix. Using PCA, we reduced this distance matrix to 10 features per TCN. These N-Orbit-derived features, along with cell-type proportions, the average Ki-67 percentage per cell type, and the treatment time point, were used as inputs for a logistic regression classifier to predict treatment outcomes from an individual TCN. Important features from this model, selected based on model coefficients and significance, were then aggregated into an image-level logistic regression classifier. Confidence scores from the classifier’s decision function were averaged to obtain an overall patient-level outcome prediction score (Fig. 4A).

CytoCommunity identified 11,871 individual TCNs across the dataset. The UMAP of the N-Orbit-based TCN distance matrix revealed distinct regions of density corresponding to TCNs from different treatment phases (Fig. 4B). N-Orbit achieved an AUROC of 0.85, higher than both the reported AUROCs in Wang et al. (C&I: 0.77, C: 0.48) and the CTE model, which omitted N-Orbit features (0.79, *p* = 0.01, Fig. 4C). These results suggest that N-Orbit-derived distances provide a substantial performance boost in predicting clinical outcomes before treatment onset. N-Orbit maintained better performance compared to CTE when generating TCNs using BANKSY and CellCharter in place of CytoCommunity (CellCharter: *p* = 0.001, BANKSY: *p* = 0.003, Supplementary Fig. 4A, B). Alternative features using dimension-reduced embeddings from BANKSY and CellCharter were also assessed on their respective generated TCNs, but optimal performance was achieved by N-Orbit on the CytoCommunity-generated TCNs.

Probability scores from the TCN-level classifier were used to identify hotspots of RD and pCR TCNs within the N-Orbit distance space. To enhance the interpretability of clinically informative N-Orbit features, we developed a bootstrap-permutation test to identify N-Orbits enriched in each TCN cluster and compiled the results into *summary graphs* based on nucleus–orbit pairs (i.e., co-memberships) (Fig. 4D–F, Supplementary Figs. 5–8).

Four RD and three pCR hotspots were identified. RD1 was centered around the AR^+^LAR malignant cell type, which is associated with immunosuppression and poor outcomes^20^. AR^+^LAR exhibited co-membership with CD15^+^ malignant cells, PDPN^+^ stromal cells, fibroblasts, and endothelial cells. Our N-Orbit analysis highlighted these relationships, building on previous findings by Wang et al. regarding CD15^+^ cells in treatment-resistant tumors. RD2 also included AR^+^LAR cell co-memberships with non-epithelial cell types and emphasized recurrent co-memberships with fibroblasts and PDPN^+^ stromal cells. pCR2 displayed recurrent co-memberships between M2 macrophages and dendritic cells, mediated by CD8^+^ GZMB^+^ and CD8^+^ PD1^+^ exhausted T cells and the absence of AR^+^LAR cells, indicating more active immune clearance in this cluster. A cell type composition-matched TCN pair highlighting increased proximity between M2 Mac and dendritic cells in a pCR2 vs. RD TCN is shown in Supplementary Fig. 9A. RD3 was centered upon Vimentin+ epithelial-to-mesenchymal transition (EMT) malignant cells with strong fibroblast association. CAFs have been implicated in driving vimentin expression and EMT in breast cancer, which is pertinent to metastasis^21,22^. Post-treatment cluster RD4 consisted of CD20^+^ B cells surrounded by various CD4^+^ and CD8^+^ T cell types, showing lymphocyte exclusivity without involvement of epithelial or stromal cells. Post-treatment cluster pCR1 displayed fibroblast-myofibroblast co-memberships, mediated by CD8^+^ T and CD56^+^ NK cells, potentially representing a stromal remodeling niche with immune surveillance. Post-treatment cluster pCR3 was centered upon basal cells and CK^hi^GATA3^+^ epithelial cells. They were associated with fibroblasts and myofibroblasts, also suggesting stromal remodeling and wound healing after treatment. Across summary graphs, most recurrent co-memberships were also preserved in the test set (Supplementary Figs. 4C, and 6). Summary graphs generated from N-Orbits derived from TCNs generated by CellCharter and BANSKY maintained similar co-memberships as well (Supplementary Figs. 4C, and 10–11). When compared with RD or pCR CytoCommunity summary graphs, Spearman correlations between maximal edge weights for cell type pairs were moderate to strong (0.573–0.889). The top 10 maximal edge-weighted cell type pairs also largely overlapped among all three TCN callers (Supplementary Figs. 18A, B).

#### Non-small cell lung cancer

**Fig. 5:**
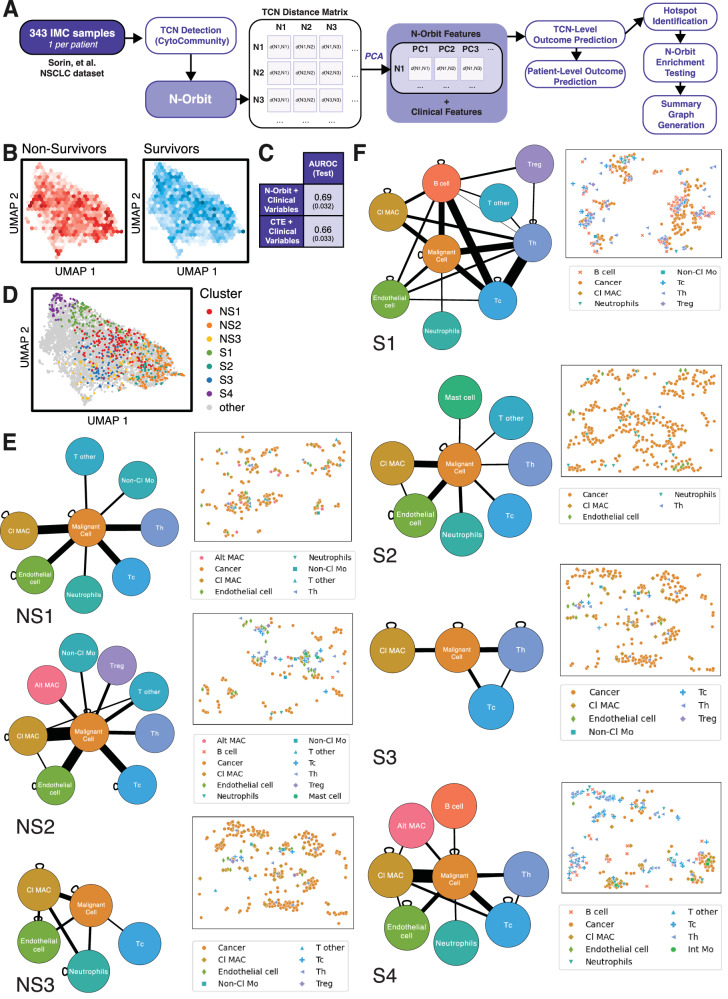
Application of N-Orbit distance to predict survival in non-small cell lung cancer (NSCLC) patients. **A** Methods schematic for patient outcome prediction using N-Orbit-derived features and the generation of summary graphs. **B** UMAP density plots for the N-Orbit-based tissue cellular neighborhood (TCN) distance matrix, faceted by treatment outcomes (Non-Survivor [NS], Survivor [S]). **C** Performance statistics for treatment outcome predictions from N-Orbit and CTE-based classifiers. Standard deviations are shown in parentheses. **D** NS and S TCN hotspots (NS1–3, S1–4) plotted on the UMAP of the TCN distance matrix. TCNs outside hotspots are plotted in gray for reference. **E** Summary graphs of NS TCN hotspots, derived from their enriched N-Orbits, with corresponding example cell type maps. Edge weights indicate the relative recurrence of cell type co-memberships among enriched N-Orbits. Self-edges indicate monotypic nucleus-orbit relationships of that cell type. **F** Summary graphs of S TCN hotspots, derived from their enriched N-Orbits, with corresponding example cell type maps. Edge weights indicate the relative recurrence of cell type co-memberships among enriched N-Orbits. Self-edges indicate monotypic nucleus-orbit relationships of that cell type. Cl MAC CD163− macrophage, Alt MAC CD163+ macrophage, Cl Mo classical monocyte, Non-Cl Mo non-classical monocyte, Int Mo intermediate monocyte, Tc cytotoxic T cell, Th helper T cell. Source data are provided as a Source Data file.

We applied a similar workflow to predict survival outcomes in patients with non-small cell lung cancer (NSCLC). We used a dataset from Sorin et al. containing 343 IMC samples, along with corresponding clinical variables and outcomes. The dataset reports 3-year survival rates for 149 survivors and 195 non-survivors^23^ (Supplementary Fig. 2E). We used CytoCommunity to generate TCNs across the dataset, which were then fed into N-Orbit to create an overall TCN distance matrix. A logistic regression classifier was trained using PCA-reduced N-Orbit distances for each TCN, along with clinical variables including sex, BMI, and smoking status. Confidence scores from the decision function of this TCN-level classifier were aggregated for each patient to generate an overall outcome prediction score (Fig. 5A).

We identified 7953 individual TCNs using CytoCommunity (Fig. 5B). N-Orbit achieved an average AUROC of 0.69 for identifying non-survivors in the test split. By contrast, an equivalent model using cell type composition instead of N-Orbit features yielded an average AUROC of 0.66 (*p* = 0.02) (Fig. 5C). When using TCNs generated by CellCharter and BANKSY, N-Orbit continued to perform better (CellCharter: *p* = 0.04, BANKSY: *p* = 0.05, Supplementary Fig. 4D). N-Orbit features also outperformed those derived from CellCharter and BANKSY embeddings on their respective TCNs (Supplementary Fig. 4E).

We generated summary graphs to characterize TCN hotspots in non-survivors (NS) and survivors (S) (Fig. 5D–F, Supplementary Figs. 12–15). Most of these graphs are centered on malignant cells. Co-memberships of malignant cells with endothelial cells, neutrophils, classical (Cl) macrophages, cytotoxic T (T_c_) cells, and helper T (T_h_) cells were also common across NS and S graphs. Notably, co-memberships between malignant cells and B-cells were only present in the S subgraphs (S1 and S3) (Fig. 5, Supplementary Fig. 15). This was consistent with the findings of Sorin et al., in which the B-cell marker, CD20, was particularly important for outcome prediction. Recurrent co-memberships among B-cells, malignant cells, and various T cell types were also present in these subgraphs. Furthermore, cliques involving malignant cells, T_c_ cells, and T_h_ cells were seen in S1, S3, and S4 subgraphs but were absent in all NS subgraphs. A cell-type composition-matched TCN pair highlighting increased proximity between T_c_ and T_h_ cells in an S vs. NS patient is shown in Supplementary Fig. 9B. These findings suggest the critical role of coordinated interactions among multiple immune cell types in combating tumor cells. Such patterns held in the test set summary graphs (Supplementary Fig. 13). Summary graphs derived using BANKSY and CellCharter as alternative TCN calling methods also had similar co-memberships (Supplementary Figs. 4F and 16–17), with strong maximal edge-weight correlations (0.716–0.817). The top 10 maximal edge-weighted cell type pairs also largely overlapped among all three TCN callers (Supplementary Figs. 18C, D).

### Trajectory and homology reconstruction using N-Orbit distances

**Fig. 6:**
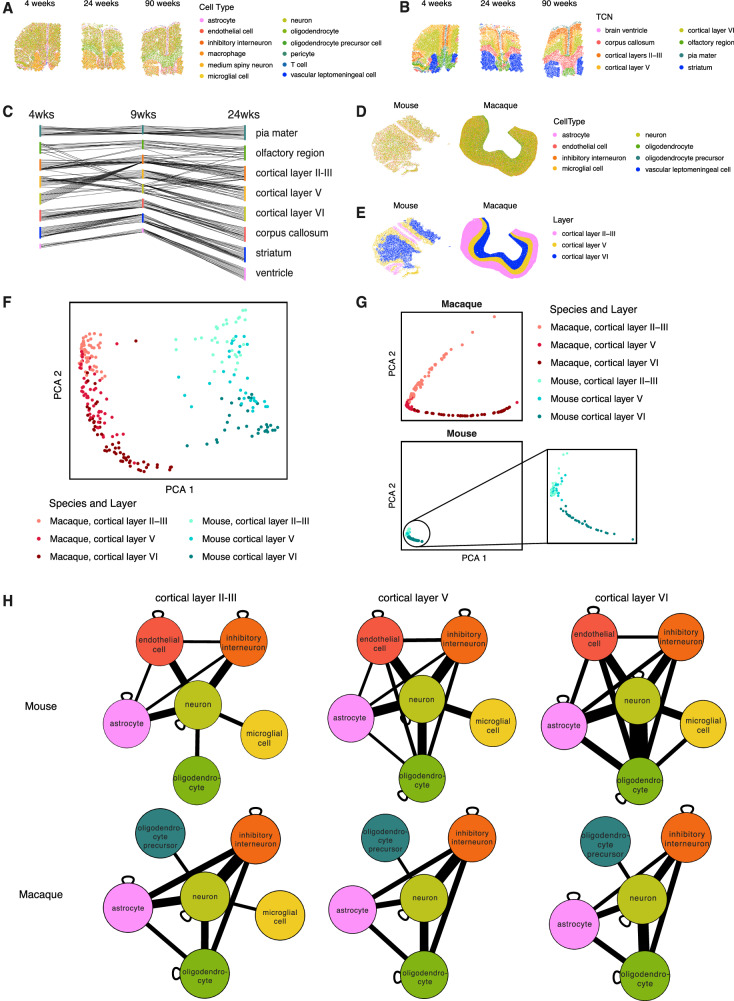
Application of N-Orbit distances to reconstruct mouse brain aging trajectories and mouse-macaque cortex homology. **A** Cell type maps of example MERFISH mouse brain samples at each age time point. **B** Tissue cellular neighborhood (TCN) maps of example MERFISH mouse brain samples at each time point. **C** Reconstructed trajectory graph of mouse brain region tissue cellular neighborhoods (TCNs) across ages, edges connecting each TCN node to the closest TCN (by N-Orbit-based TCN distance) in the adjacent time point. **D** Cell type maps of example frontal cortex samples from mouse (MERFISH) and macaque (Stereo-seq). **E** TCN maps of example frontal cortex samples from mouse (MERFISH) and macaque (Stereo-seq).** F** PCA plot of the N-Orbit-based TCN distance matrix for mouse and macaque cortex, color-coded by species and cortex layer. **G** PCA plots of CTE-based TCN distance matrix from mouse and macaque cortices, color-coded by species and cortex layer, including a zoomed-in view of mouse cortical TCNs. **H** Summary graphs of each species and cortex layer, derived from enriched N-Orbits. Edge weights indicate the relative recurrence of cell type co-memberships among enriched N-Orbits. Self-edges indicate monotypic nucleus–orbit relationships of that cell type. Source data are provided as a Source Data file.

We utilized N-Orbit to identify analogous brain TCNs across time in the aging mouse brain. The dataset from Allen et al. includes 31 MERFISH samples from the frontal cortex and striatum of 12 mice across 3 ages, with corresponding cell-type and brain-region annotations (Fig. 6A, B)^24^. Manually annotated brain regions served as TCNs, totaling 241 across all samples. Using N-Orbit distances, we constructed a multipartite graph to visualize connections among TCNs across the three ages (Fig. 6C). Our analysis revealed strong continuity of connections between TCNs representing the same brain region over time, with cross-regional connections occurring only within the cortical and olfactory layers.

We examined whether N-Orbit could capture homology between analogous regions across different species (Fig. 6D, E) by integrating a macaque cortex dataset from Chen et al. with a mouse brain dataset from Allen et al., focusing on frontal cortex layers^25^. The Chen et al. dataset includes 51 Stereo-seq samples spanning slices from a single macaque brain. Across both datasets, there were 246 TCNs annotated by their respective frontal cortex layers. We projected the TCN distance matrix for both datasets using PCA (Fig. 6F). The first principal component distinguished species, while the second strongly corresponded to the cortex layer, forming an ordered pattern across both species. This layer-specific pattern was not observed with CTE-based distances (Fig. 6G). Summary graphs indicated consistent cell-type membership within each species and variable cell-type co-membership across cortical layers (Fig. 6H). Notably, neuron-oligodendrocyte co-memberships increased in deeper layers in both the mouse and macaque samples.

## Discussion

We present a mathematical model and associated metric, N-Orbit, that enables principled comparison of TCNs. Unlike methods that rely solely on cell-type composition and enrichment, N-Orbit can capture complex relationships among cell types within a TCN. In addition, N-Orbit provides better practicality compared to subgraphs, due to the consideration of only proximity between cell types rather than the exact subgraph topology. This mathematical construct also allows for a vector representation of the N-Orbit structure, facilitating scalable computation of N-Orbit and TCN distances. Our results demonstrate that N-Orbit effectively captured variations in spatial organization across diverse tissue types, outperforming cell-type enrichment and graph-embedding-based methods. Our findings suggest that N-Orbit is broadly applicable across biological contexts, including healthy, aging, and diseased tissues, as well as homologous structures across different species.

N-Orbit’s distance metric significantly broadens the scope of TCN analyses on spatial omics data. For TCN detection methods that cannot perform multisample alignment, this metric enables clustering and the identification of recurring TCNs across datasets. It allows projection of TCNs onto a common coordinate space and detection of patterns associated with temporal and clinical variables. The N-Orbit structure is highly generalizable, requiring only discrete units (e.g., cells) with spatial and phenotypic information. Consequently, N-Orbit can be applied to diverse tasks, ranging from examining spatial organizational changes during embryological development to tracking the evolution of TCN-level structures within the tumor microenvironment. Lastly, because our TCN distance metric is derived from a well-defined N-Orbit structure, TCNs and TCN clusters of interest can be traced back to their characteristic N-Orbits, allowing for interpretability.

### Further potential applications

We primarily focused on N-Orbit applications that could be quantitatively assessed against established methods and on common benchmarking datasets. We envision the bread-and-butter applications to include integrating and clustering TCNs across a dataset, either through unsupervised dissection of the TCN space or supervised hotspot detection, followed by interpretation via enrichment testing and summary graph generation. N-Orbit could also be applied to a wide array of analyses beyond the examples in this paper. For example, many well-established TCN detection methods, such as GraphST, either do not support multi-sample alignment or require excessive memory for processing large datasets. N-Orbit performs post hoc alignment to identify analogous TCNs across samples. Furthermore, because N-Orbit operates on cell phenotype rather than expression data, it could facilitate spatial meta-analyses across datasets of different modalities and diverse tissues. Integration of independent datasets with N-Orbit could help construct large-scale spatial reference atlases, such as a pan-cancer atlas. As a distance metric, N-Orbit could also be used to quantify spatial heterogeneity within tissues, akin to diversity indices in ecology. Overall, N-Orbit provides a quantitative means of comparing spatial organization, which can be flexibly applied to derive qualitative insights.

### Limitations

N-Orbit requires single-cell resolution spatial omics data with annotated cell types. Because it relies on nearest-neighbor distance to characterize spatial proximity between cell types, it is not applicable to spot-level spatial omics data. N-Orbit also does not generate TCNs de novo and thus depends on external TCN detection methods, which must be performed upstream. As a result, the biological relevance of N-Orbit features depends on the quality of results from the upstream TCN detection method. The quality and granularity of cell type annotations may also influence performance, though N-Orbit performed well across datasets ranging from 15 cell types in the hypothalamus dataset to 47 in the pulmonary fibrosis dataset. While we observed substantial overlap in biological findings among TCN callers, there was still some variation. An inappropriate choice of the TCN caller may lead to missed or false-positive discoveries. The accuracy of the TCNs generated by the upstream caller is of utmost importance to N-Orbit’s utility. Across our analysis, we found several examples of CellCharter showing better concordance with ground truth through both N-Orbit-independent (e.g., AMI) and N-Orbit-dependent (e.g., AUROC) metrics. We also observed greater overlap in biological findings between CellCharter and CytoCommunity than with BANKSY, especially for the TNBC pCR TCNs. In addition to biological accuracy, TCNs should exhibit some degree of spatial coherence to avoid overly sparse N-Orbit structures and edge effects. Consequently, N-Orbit is more compatible with TCN detection methods that emphasize spatial coherence, such as graph neural network-based methods. As a result, we generally recommend CellCharter and CytoCommunity for use with N-Orbit.

## Methods

### Inputs

N-Orbit computes a post hoc, alignment-free distance metric for TCNs generated by a separate upstream method (e.g., manual annotation, CytoCommunity, and CellCharter). A tabular input containing sample names, coordinates, cell types, and TCN assignments for each cell is required.

### N-Orbit construction

We started with single-cell-resolution spatial omics data, in which each cell had an assigned cell type and spatial coordinates measured in microns. To decompose a TCN into its constituent N-Orbits, we first constructed an *r*-radius graph based on spatial coordinates, with nodes labeled by cell types. When TCNs were the unit of interest, this graph was filtered to include only nodes corresponding to cells within each TCN. If samples were the unit of interest, this filtering step was omitted. For each cell *a* in the graph, we calculated a proximity score to each adjacent cell *b* based on their physical distance using the following method:

1 $${{\mbox{Proximity}}}\left(a,b\right)=\,\frac{10}{\min (\max \left({{\mbox{dist}}}\left({{a}},{b}\right),10\right),r)}$$

### Vector representation of N-Orbits

We represented N-Orbits as vectors for computational analysis. This vector representation had two components: a nucleus encoding and an orbit encoding. Each component had an element for each possible cell type in the dataset, with indices arranged alphabetically. The nucleus encoding was set to zeros for all indices, except at the one corresponding to the center cell type, which was assigned a parameter value, *p*, described in the *Distance between N-Orbits* section. The orbit encoding contained the maximal proximity score (derived from the shortest distance) for each possible cell type. If there were no cells of a certain type within the search radius, the value was set to 0.

### Distance between N-Orbits

The distance between a pair of N-Orbits was calculated as the Manhattan distance between their vector representations, representative of an edit distance. The nucleus change penalty parameter, *p*, determined the cost of changing the center cell type and can be adjusted to be higher than the cost of changing cell types within the orbits.

### Calculation of N-Orbit-based TCN and sample distance

The overall distance between two TCNs (or samples) was calculated as the minimum cost to convert the set of N-Orbits representing the first TCN (or sample) to the set of N-Orbits representing the second TCN (or sample). To ensure sets of the same size, we used a bootstrap of N-Orbits with size *s* for each TCN (or sample). A cost matrix optimization using SciPy’s Linear Sum Assignment function was used to compute the minimum distance between these two sets of N-Orbit vectors^26^. Let $D$ be an *s *× *s* distance matrix between the N-Orbits representing the two TCNs. Let $X$ be an *s*×*s* Boolean matrix representing a bijection between the N-Orbits of the two TCNs, where ${X}_{{ij}}=1$ if the *i*-th N-Orbit in the first TCN was mapped to the *j*th N-Orbit in the second TCN. The pairwise TCN distance was given by

2 $$\min {\sum}_{i}{\sum}_{j}{D}_{{ij}}{X}_{{ij}}$$

Distances for every possible pair of TCNs (or samples) were compiled into an overall TCN (or sample) distance matrix. Only TCNs of at least 40 cells were considered. To account for the N-Orbit sample size, distances were scaled by dividing by *s*.

### Calculation of cell type enrichment-based TCN and sample distances

We developed a similar schema for calculating TCN and sample distance based solely on cell type enrichment. The negative base-10 logarithms of *p*-values from one-sided hypergeometric testing for each possible cell type in a TCN (or sample) were compiled into a *cell type enrichment vector*. For *p*-values below the minimum value representable in Python (1e − 309), the negative log p-values were recorded as 309. The distance between TCNs (or samples) was then calculated as the Manhattan distance between their respective cell type enrichment vectors.

### Calculation of density-based TCN distances

We compared our N-Orbit vector formulation, which depends on nearest-neighbor distances, with one that instead uses density for each cell type. Instead of the orbit encoding, this vector contained the number of cells of each cell type within the specified radius, including the center cell. No nucleus encoding was included in this vector formulation. Specifically, if *n* is the number of unique cell types in a dataset, each cell *c* corresponds to a density vector *v* of length *n* where *v*_*i*_ is the frequency of cell type *i* within radius *r* of cell *c*. Downstream vector distance and neighborhood distance calculation procedures were kept the same as the typical N-Orbit-based distance calculation, as described in *Distance between N-Orbits* and *Calculation of N-Orbit-based TCN and sample distance* sections.

### Synthetic TCN generation

Spatial coordinates for cells in the synthetic TCNs were generated using a Poisson process model (Fig. 2A–E, Supplementary Fig. 1A–D). TCNs were assigned to cells by seeding one cell representing each TCN at random and allowing TCNs to expand along a 6-Nearest Neighbors spatial graph. The rate of spread was proportional to the pre-specified TCN composition within the sample. Following the TCN assignment, cell type assignments were generated using a pairwise Markov random field^27^. This method parameterizes the joint distribution of the labeled 6-Nearest TCN graph based on node potentials ${{{\rm{\phi }}}}\left({x}_{i}\right)$ and edge potentials ${{{\rm{\psi }}}}\left({x}_{i},{x}_{j}\right)$, where ${x}_{i}$ denotes the cell type of node $i$. The joint distribution for $x=\left\{{x}_{1},\cdots,{x}_{n}\right\}$ was given by

3 $$P\left(x\right)=\frac{1}{z}\exp \bigg({\sum}_{i\,\in \,V}{{{\rm{\phi }}}}\left({x}_{i}\right)+{\sum}_{\left(i,j\right)\,\in \,E}{{{\rm{\psi }}}}\left({x}_{i},{x}_{j}\right)\bigg)$$

where $z$ is a normalization constant that ensures the probability distribution sums to 1, and *V* and *E* are the sets of graph vertices and edges, respectively. In cases where cell types were independently distributed due to uniform edge potentials, the relative cell type proportions, or marginal distributions, were determined by the SoftMax function of the node potentials

4 $$M\left({{{\boldsymbol{x}}}}\right)=\frac{1}{z}\exp \bigg({\sum}_{i}\phi \left({x}_{i}\right)\bigg)$$

Cell type labels in the graph were initialized according to this marginal distribution. After initialization, cell types were resampled in random order according to the joint distribution.

To estimate the proper node potentials that would yield the target cell type composition ${M}_{T}$ with the specified edge potential, node potentials were adjusted iteratively until the target parameters were approximated, as follows.

1. Node potentials were initialized as the logit transformation of the target marginal.
2. A sample was generated according to the specified edge potentials $\psi \left({x}_{i},{x}_{j}\right)$, and current node potentials ${\phi }_{k}\left({x}_{i}\right)$.
3. The empirical marginal distribution of the sample ${M}_{e}$ was calculated along with its difference from the target marginal $\Delta M={M}_{e}-{M}_{T}$.
4. The new node potential was formulated based on $\Delta M$ and a pre-specified learning rate ${{{\rm{\alpha }}}}=\,0.1$.
  5 $${{{{\rm{\phi }}}}}_{k+1}\left({x}_{i}\right)={{{{\rm{\phi }}}}}_{k}\left({x}_{i}\right)-{{{\rm{\alpha }}}}{\left|\Delta M\right|}^{1.25}\Delta M$$
5. This process was repeated until either $\left|\Delta M\right| < 0.03$ or 1000 iterations were completed.

Once the proper node and edge potentials were approximated for each TCN, these parameters were used to generate the specified number of synthetic samples. For each of the two simulated experiments, 50 samples—each consisting of roughly 10,000 cells—were generated for each sample type.

### Comparing distance metrics with AUROC

The ability of the N-Orbit and CTE distance metrics to distinguish between two groups (e.g., similar vs. dissimilar TCNs) was assessed based on their performance as single-variable classifiers, as measured by the area under the receiver operating characteristic curve (AUROC). The AUROC value was calculated from the true and false positive rates (TPR and FPR) at various thresholds, as follows:

6 $${\mbox{AUROC}}={\int }_{0}^{1}{\mbox{TPR}}\left({\mbox{FPR}}\right)d{\mbox{FPR}}$$

To compare the ROC curves and assess significant differences in performance between N-Orbit and CTE distance-based classifiers, we used the one-sided DeLong test (from the *pROC* R package), along with the corresponding *p*-value. In some cases, the tests yielded p-values below the minimum representable number in *R*, which were recorded as *p* < 1e−324.

### Running of published methods

We evaluated N-Orbit’s performance on TCNs generated by BANKSY and CellCharter for the CODEX spleen, MERFISH hypothalamus, Xenium pulmonary fibrosis, IMC TNBC, and IMC NSCLC datasets, as well as on CytoCommunity for the latter two datasets. In addition, we compared N-Orbit-derived features with embedding features from BANKSY, CellCharter, SpaGCN, and GraphST on the CODEX spleen, MERFISH hypothalamus, and Xenium pulmonary fibrosis datasets; we also compared N-Orbit with features from BANKSY and CellCharter for the IMC TNBC and IMC NSCLC datasets. For BANKSY, CellCharter, SpaGCN, and GraphST, cell gene expression and spatial coordinates were used as inputs. For CytoCommunity, cell-type annotations and spatial coordinates served as inputs. For TCN generation on the spleen, hypothalamus, and pulmonary fibrosis datasets, a range of hyperparameter combinations was tested, as shown in Supplementary Fig. 3. Otherwise, default hyperparameters were primarily used, except for the number of TCNs, which was set to most closely match TCNs with manual annotations based on AMI when available. The Python versions of each package (BANKSY v1.3.3, CellCharter v0.3.3, SpaGCN, GraphST v1.0.0, CytoCommunity2) were used.

### TCN instances in CODEX mouse spleen data

TCNs in the mouse spleen CODEX dataset were divided into TCN *instances* by constructing a 50-µm spatial graph with nodes labeled by TCN type. For each TCN, the graph was then filtered to include only nodes with the corresponding label, and each resulting connected component with at least 40 cells was designated as a separate TCN instance.

### TCN instances in the mouse hypothalamus MERFISH data

Taking advantage of the bilaterally symmetric anatomy, we divided non-midline structures into left and right TCN instances, resulting in 101 instances across the dataset (Supplementary Fig. 2B). For each sample, an *x*-axis threshold was manually determined to split each sample into left and right halves. For all non-midline structures, cells within each hypothalamic nucleus region on either side of the threshold were designated as separate TCN instances.

### TCN detection in TNBC and NSCLC datasets

Supervised CytoCommunity, using the treatment phase as sample labels, was used to generate TCNs from the TNBC dataset, with a maximum of 10 TCNs per sample. Unsupervised CytoCommunity was used to generate TCNs from the NSCLC dataset, with a maximum of 10 TCNs per sample. CellCharter and BANKSY were also used to generate alternate TCNs and embeddings.

### TNBC treatment response prediction models

An initial Logistic Regression model with L1 regularization was trained to predict clinical outcomes from features of individual TCNs. Input features were derived from rows of the TCN distance matrix reduced via PCA (# components = 10), cell type proportions, and Ki-67 percentages per cell type. Each row of the TCN distance matrix represented a vector of distances from one TCN to all other TCNs in the dataset. Separate features were created for each of the three treatment phases (Baseline, On-Treatment, Post-Treatment) for these variables, with values set to 0 if the TCN was derived from a different treatment phase. These features were *Z*-score transformed. Additional indicator features for the treatment phase were also included. Patients were designated into four splits, with three splits used for feature selection and validation at a time, and the remaining split reserved for independent testing. Samples from underrepresented classes were given greater weight in the loss function, ensuring that all classes had balanced weights overall. Important features were selected based on the significance and magnitude of the coefficients of the fitted classifier (Bonferroni-adjusted two-sided Z-test *p*-value < 0.05, magnitude > 0.075). For image-level predictions, TCNs within each image were ranked based on the proportion of cells in that image belonging to that TCN in descending order. A feature was created for each important feature of each TCN rank (e.g., PC1 of the Rank 1 TCN). Ranks past the number of TCNs in an image had feature values imputed as the median. An equivalent Logistic Regression classifier was trained on these image-level features. For final patient-level predictions, decision-function-derived confidence scores from the image-level classifier were averaged across the Baseline timepoint images for that patient. For the CTE-only version of the model, N-Orbit-derived PCA features were omitted, and all other steps remained the same. Paired t-tests (one-sided) were used to assess significant differences in performance between N-Orbit and CTE versions across test splits.

### NSCLC 3-year survival prediction models

Similar to the TNBC model, the NSCLC prediction model used a Logistic Regression classifier with L1 regularization, trained on PCA-reduced N-Orbit distances (# components = 40), along with cell type proportions and clinical variables (sex, BMI, smoking status, pack years, and stage). Features were Z-score transformed, class weights were balanced, and patients were split into fourfolds for cross-validation and hold-out testing. Features were selected based on the significance and magnitude of coefficients in the fitted classifier (Bonferroni-adjusted two-sided Z-test *p*-value < 0.05; magnitude > 0.075), and the classifier was retrained on these features. Patient-level predictions were aggregated from the TCN-level confidence scores of this final model. In the CTE-only version of the model, only cell-type proportion and clinical-variable features were used. Paired *t*-tests (one-sided) were used to assess significant differences in performance between N-Orbit and CTE versions across test splits.

### Benchmarking against CellCharter and BANKSY embeddings for TNBC and NSCLC datasets

Because CellCharter and BANKSY generate cell-level rather than TCN-level embeddings, we averaged cell embeddings across TCNs for direct comparison. For both datasets and methods, the averaged embeddings were reduced using PCA with the same number of components used in the N-Orbit versions. These reduced features replaced N-Orbit-derived features, while all other variables and procedures were kept the same to assess comparative performance.

### Identifying TCN hotspots of interest

Groups of correctly predicted TCNs with high probability (>0.75 for TNBC, >0.65 for NSCLC) for their respective outcome (e.g., residual disease, pathological complete response for the TNBC dataset) were clustered into hotspots using Leiden clustering on the N-Orbit-derived distances. Clusters of sufficient size were denoted “hotspots” and retained for further analysis. The last of the four patient splits was designated as the test set, for which clustering was performed independently.

### N-Orbit enrichment test

The sampled N-Orbit vectors generated during the TCN distance calculation were used to identify enriched N-Orbits in the TCN hotspots of interest, ensuring equal representation of all TCNs in the cluster. N-Orbit vectors were converted into binary versions based on a proximity score threshold of 0.2 (equivalently 50 µm) so that repeating structures could be identified. Enrichment was determined using a series of bootstrap permutation tests. For each test, 20% of the N-Orbit vectors were bootstrapped from the overall set and compiled into a matrix, with rows corresponding to N-Orbit vectors. The nucleus encoding portions remained fixed, while the indices of the non-zero values within the orbit encoding portions were permuted. This permutation procedure approximately preserved the marginal distribution of cell types and the distribution of N-Orbit cardinalities. For each unique N-Orbit in the original set, the occurrences in the unpermuted and permuted bootstraps were compared. Across 5 million tests, the proportion of tests where the count of the N-Orbits in the unpermuted bootstrap exceeded that in the permuted bootstrap was recorded as the *p*-value. Multiple testing correction was performed using the Benjamini–Hochberg method.

### Summary graph generation

Summary graphs were generated based on the set of enriched N-Orbits. A node was included for each cell type involved in any of the enriched N-Orbits. Solid edges were drawn for all nucleus-orbit relationships in the enriched set, weighted by the number of appearances across the N-Orbits. Self-edges were drawn to indicate monotypic relationships of each cell type. Summary graphs were pruned if they had ten or more nodes. Pruned summary graphs were generated by filtering to include nodes associated with edges of weight greater than one and nodes participating in cliques of size three or larger. All visualizations were created using Cytoscape^28^.

### Reporting summary

Further information on research design is available in the Nature Portfolio Reporting Summary linked to this article.

## Supplementary information

Supplementary Information

Reporting Summary

Transparent Peer Review file

## Data Availability

Mouse spleen CODEX dataset is available at 10.1016/j.cell.2018.07.010. Mouse hypothalamus MERFISH dataset is available at 10.1126/science.aau5324. Human pulmonary fibrosis Xenium dataset is available at 10.1016/j.cell.2024.03.013. Human TNBC IMC dataset is available at 10.1038/s41588-025-02080-x. Human NSCLC IMC dataset is available at 10.1038/s41586-022-05672-3. Mouse brain MERFISH dataset is available at 10.1016/j.cell.2022.12.010. The macaque brain Stereo-Seq dataset is available at 10.1016/j.cell.2023.06.009. Source data are provided with this paper at Zenodo and are accessible via 10.5281/zenodo.19825245 [https://zenodo.org/records/19825246].

## Code availability

The code used to develop the model, perform the analyses, and generate results in this study is publicly available on GitHub at https://github.com/tanlabcode/N-Orbit under the MIT license. The specific version of the code associated with this publication is accessible at https://zenodo.org/records/19742192 via the DOI doi:10.5281/zenodo.19742192^29^.
