# Supplementary material for: N-Orbit: towards a universal model and metric for comparing tissue microenvironments
Source: Nat Commun. 2026 May 22;17:6741. doi: 10.1038/s41467-026-73561-8 (PMC13385583; doi:10.1038/s41467-026-73561-8)
Supplement: Supplementary file 1 — Supplementary Information [file 41467_2026_73561_MOESM1_ESM.pdf]

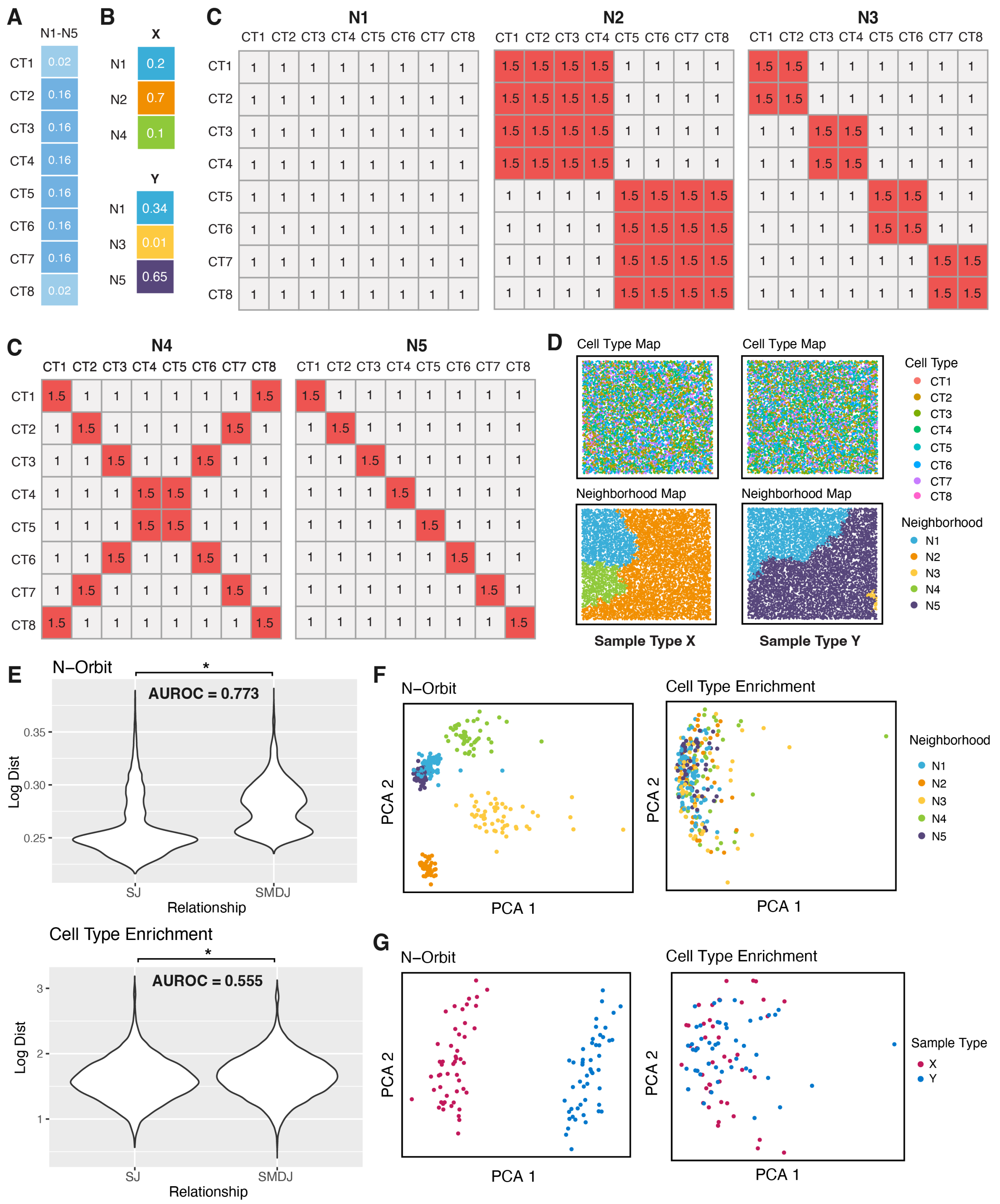

Supplementary Figure 1: Performance evaluation of the N-Orbit model to distinguish synthetic tissue cellular neighborhoods (TCNs) of similar marginal but different joint distributions (Experiment 2). A) Cell type (CT) compositions for each TCN type N1-N5. **B)** Proportions of each TCN within sample types X and Y. **C)** Cell type interactions (edge potentials) for each TCN. **D)** Example cell type and TCN maps for sample types X and Y. **E)** Violin plots of log (scaled) N-Orbit and Cell Type Enrichment (CTE)-based TCN distances for TCN pairs of same joint (SJ), same

marginal but different joint (SMDJ) distributions. Asterisks indicate statistical significance by a one-sided t-test. The area under the receiver operating characteristic curve (AUROC) is provided for the SJ vs. SMDJ comparison. **F)** PCA plots of N-Orbit and CTE TCN distance matrices, color-coded by TCN. Each point represents one TCN. **G)** PCA plots of N-Orbit and CTE sample distance matrices, color-coded by sample type. Each point represents one sample. Source data are provided as a Source Data file.

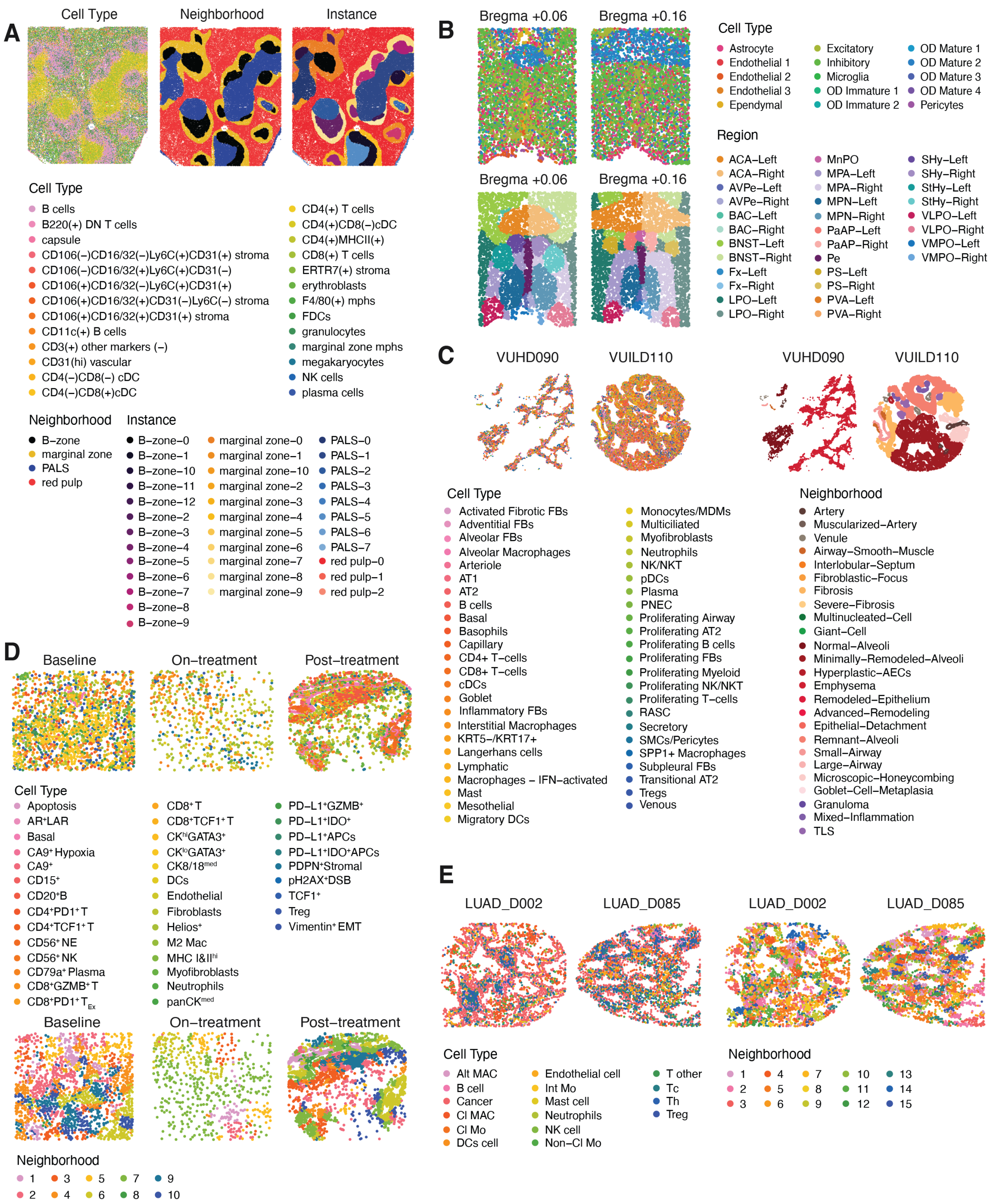

Supplementary Figure 2: All cell type and tissue cellular neighborhood (TCN) maps for datasets not included in the main figures. **A**) Cell type, TCN, and TCN instance maps for one mouse spleen CODEX sample. **B**) Cell type and TCN maps for two mouse hypothalamus MERFISH samples. **C**) Cell type and TCN maps for two human pulmonary fibrosis Xenium samples. **D**) Cell type and TCN maps for IMC samples from each treatment phase (Baseline, On-treatment, Post-treatment) for one TNBC patient. **E**) Cell type and TCN maps for two human non-small cell lung cancer imaging mass cytometry (IMC) samples.

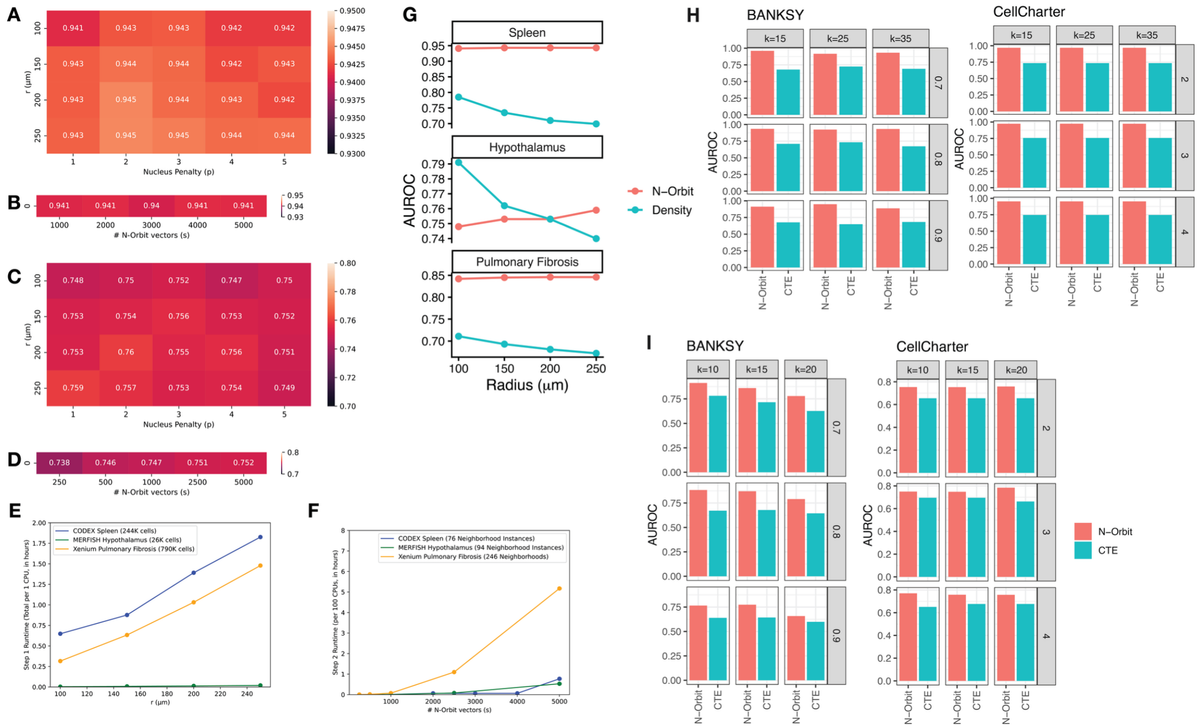

Supplementary Figure 3: Hyperparameter, robustness, and runtime testing for the N-Orbit model. **A**) AUROCs across hyperparameters for nucleus penalty *p* and search radius *r* for the CODEX mouse spleen dataset. **B**) AUROCs across parameters for the vector sample size *s* for the CODEX mouse spleen dataset. **C**) AUROCs across hyperparameters for nucleus penalty *p* and search radius *r* for the MERFISH mouse hypothalamus dataset. **D**) AUROCs across parameters for the vector sample size s for the MERFISH mouse hypothalamus dataset. **E**) Runtime for Step 1 (N-Orbit enumeration) in relation to search radius *r* for three benchmarking datasets. **F**) Runtime for Step 2 (distance calculation) in relation to vector sample size *s* for three benchmarking datasets. **G)** Comparison to a density-based alternative vector and distance formulation across various radii on the spleen, hypothalamus, and pulmonary fibrosis datasets. **H)** Hyperparameter testing for N-Orbit on BANKSY (left) and CellCharter (right) TCNs on the spleen dataset. Facet columns are kNN parameters for both BANKSY and CellCharter. Facet rows are the lambda parameter for BANKSY and the number of graph neural network layers for CellCharter. **I)** Hyperparameter testing for N-Orbit on BANKSY (left) and CellCharter (right) TCNs on the hypothalamus dataset. Facet columns are kNN parameters for both BANKSY and CellCharter. Facet rows are the lambda parameter for BANKSY and the number of graph neural network layers for CellCharter. Source data are provided as a Source Data file.

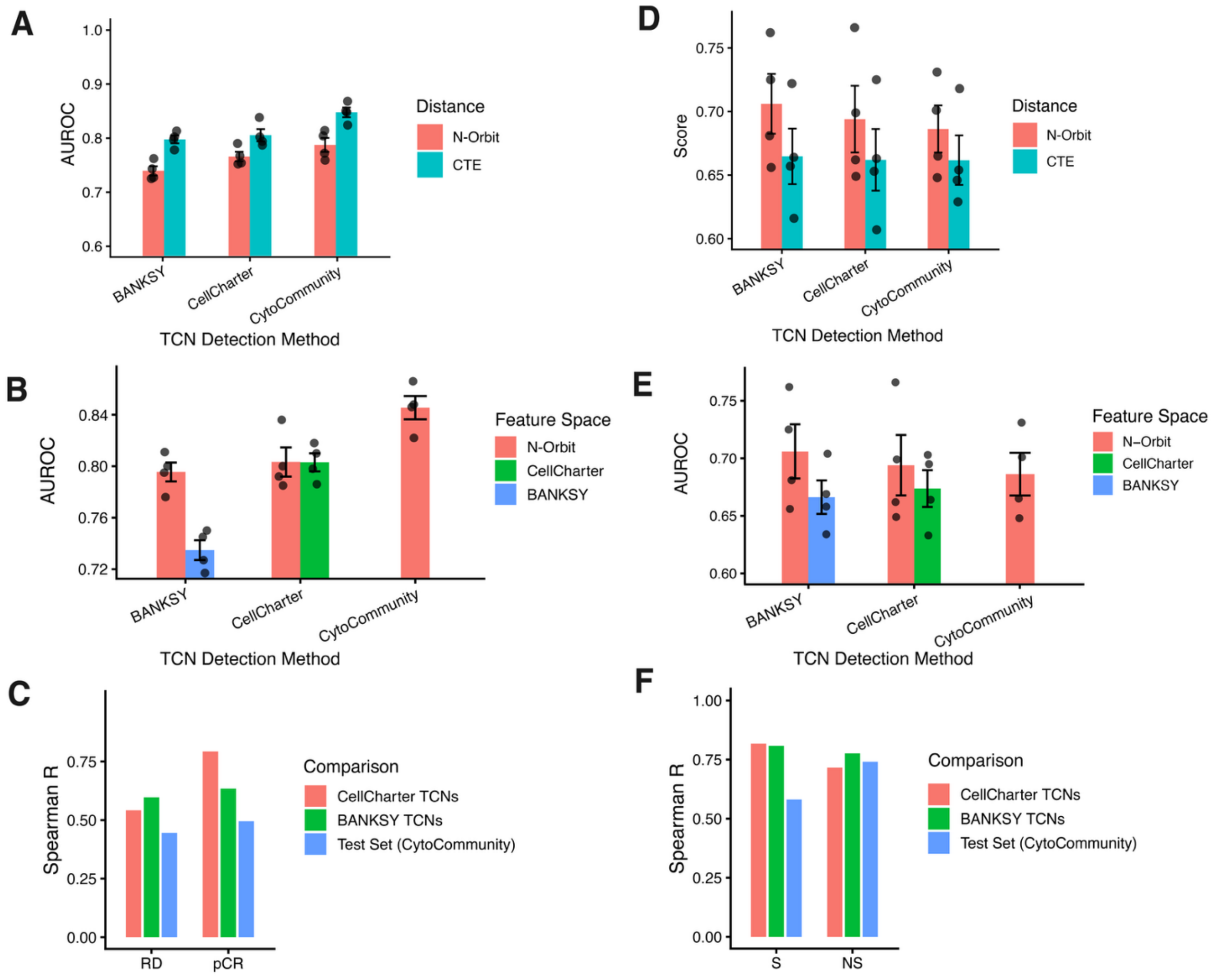

Supplementary Figure 4. Additional benchmarking for triple-negative breast cancer (TNBC) and non-small cell lung cancer (NSCLC) datasets. A) TNBC outcome prediction performance on TCNs generated using alternative methods. Error bars denote standard deviation. **B**) TNBC outcome prediction performance on the alternative feature space. Error bars denote standard deviation. **C)** Spearman correlation between maximal RD and pCR summary graph edge weights using CellCharter (training set), BANKSY (training set), and CytoCommunity (test set), compared with CytoCommunity (training set) on the TNBC dataset. Error bars denote standard deviation. **D**) NSCLC outcome prediction performance on TCNs generated using alternative methods. Error bars denote standard deviation. **E)** NSCLC outcome prediction performance on the alternative feature space. **F)** Spearman correlation between maximal S and NS summary graph weights using CellCharter (training set), BANKSY (training set), and CytoCommunity (test set), compared with CytoCommunity (training set) on the NSCLC dataset. Source data are provided as a Source Data file.

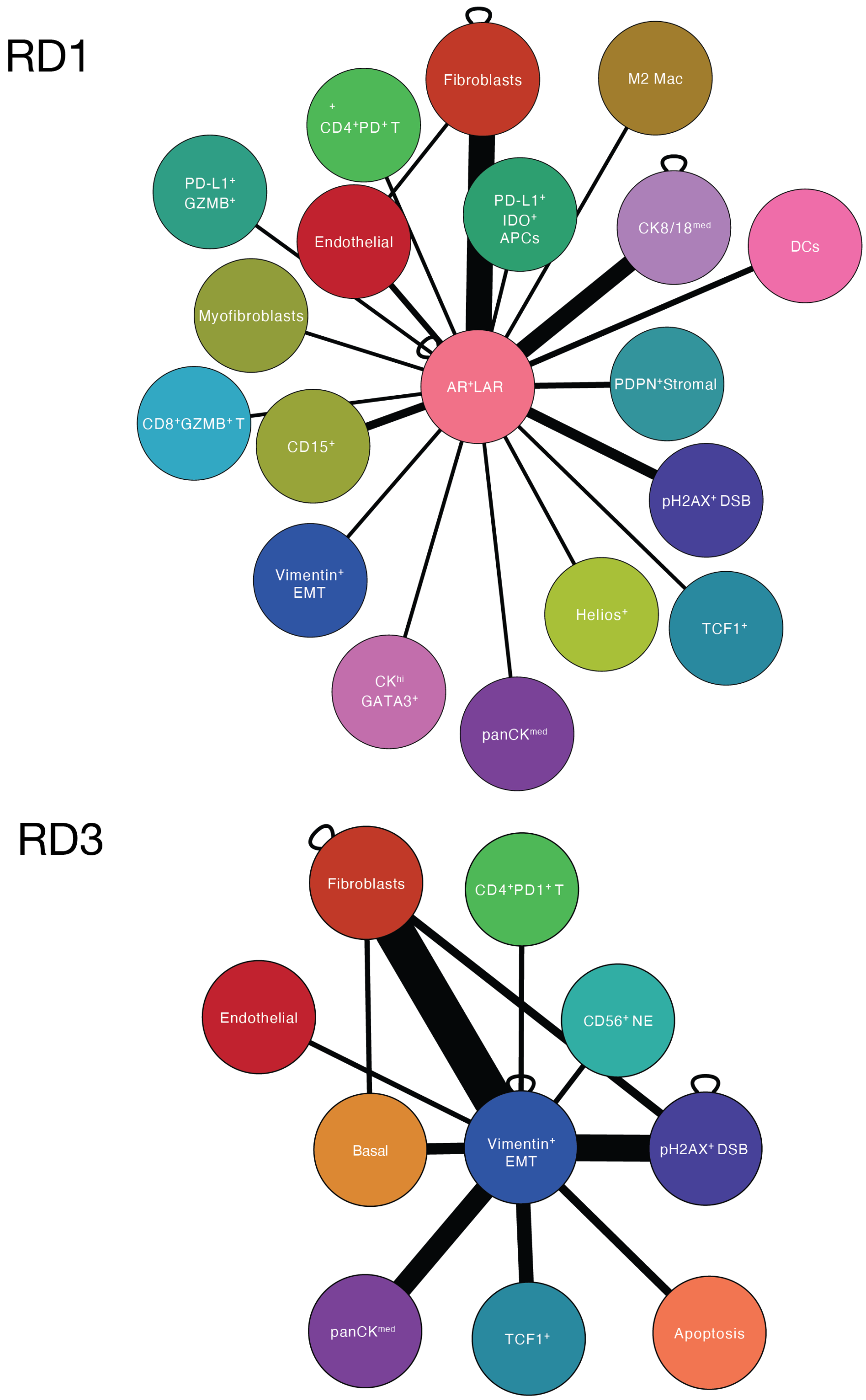

Supplementary Figure 5: Full summary graphs for tissue cellular neighborhood (TCN) hotspots from the Wang *et al*. triple-negative breast cancer (TNBC) dataset that were pruned in the main figure. Edge weights indicate the relative recurrence of cell type co-memberships among enriched N-Orbits. Self-edges indicate monotypic nucleus-orbit relationships of that cell type.

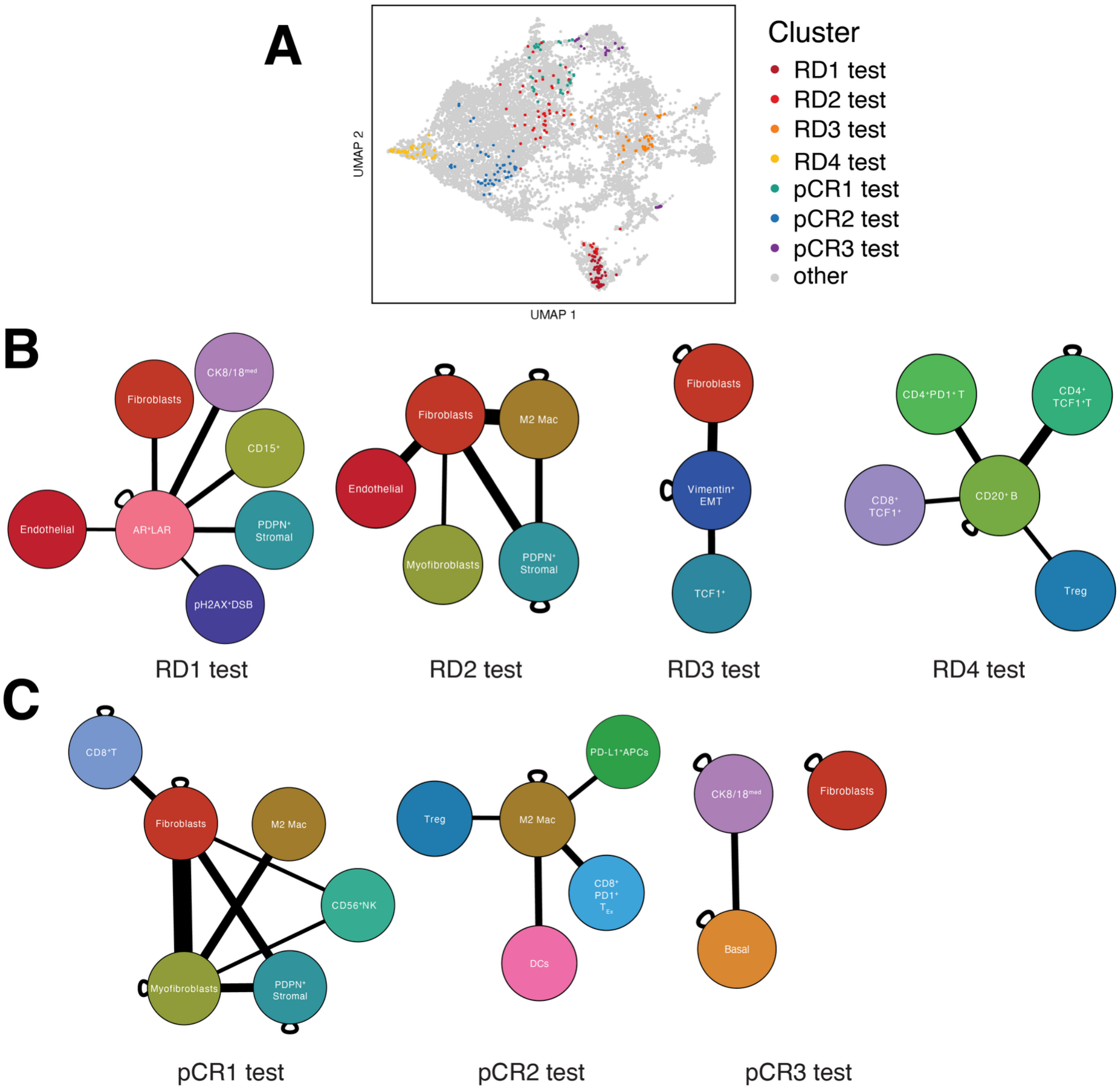

Supplementary Figure 6: Test set summary graphs for tissue cellular neighborhood (TCN) hotspots from the Wang *et al*. TNBC dataset. A) Residual disease (RD) and pathological complete response (pCR) TCN hotspots plotted on the UMAP of the TCN distance matrix. TCN outside hotspots were plotted in gray for reference. **B-C**) Summary graphs. pCR 2 and 3 did not have any enriched N-Orbits after Benjamini-Hochberg correction, so an unadjusted p-value of 0.05 was used for these graphs. Source data are provided as a Source Data file.

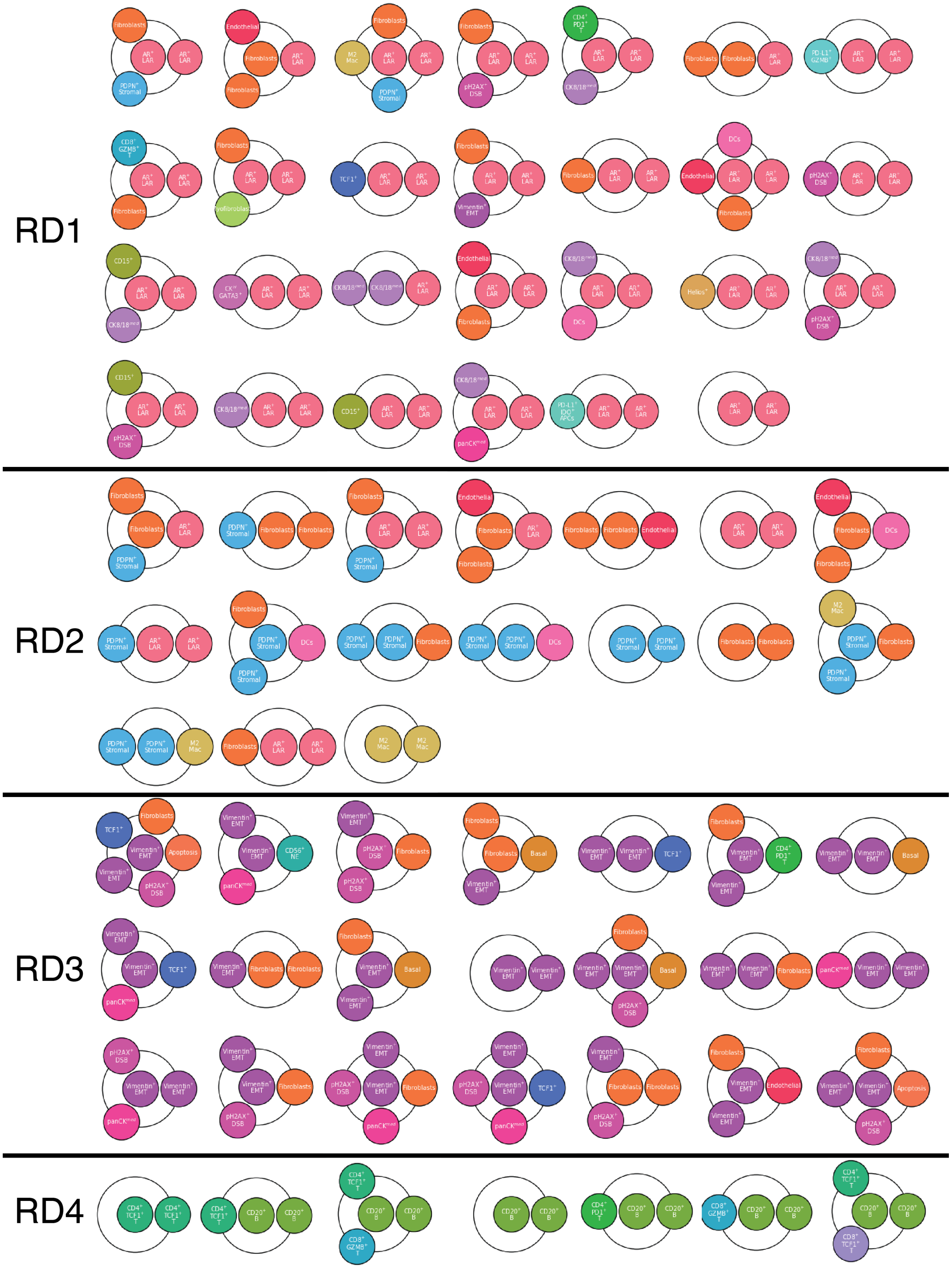

Supplementary Figure 7: List of enriched N-Orbits from the residual disease (RD) tissue cellular neighborhood (TCN) hotspots from the Wang *et al*. TNBC dataset.

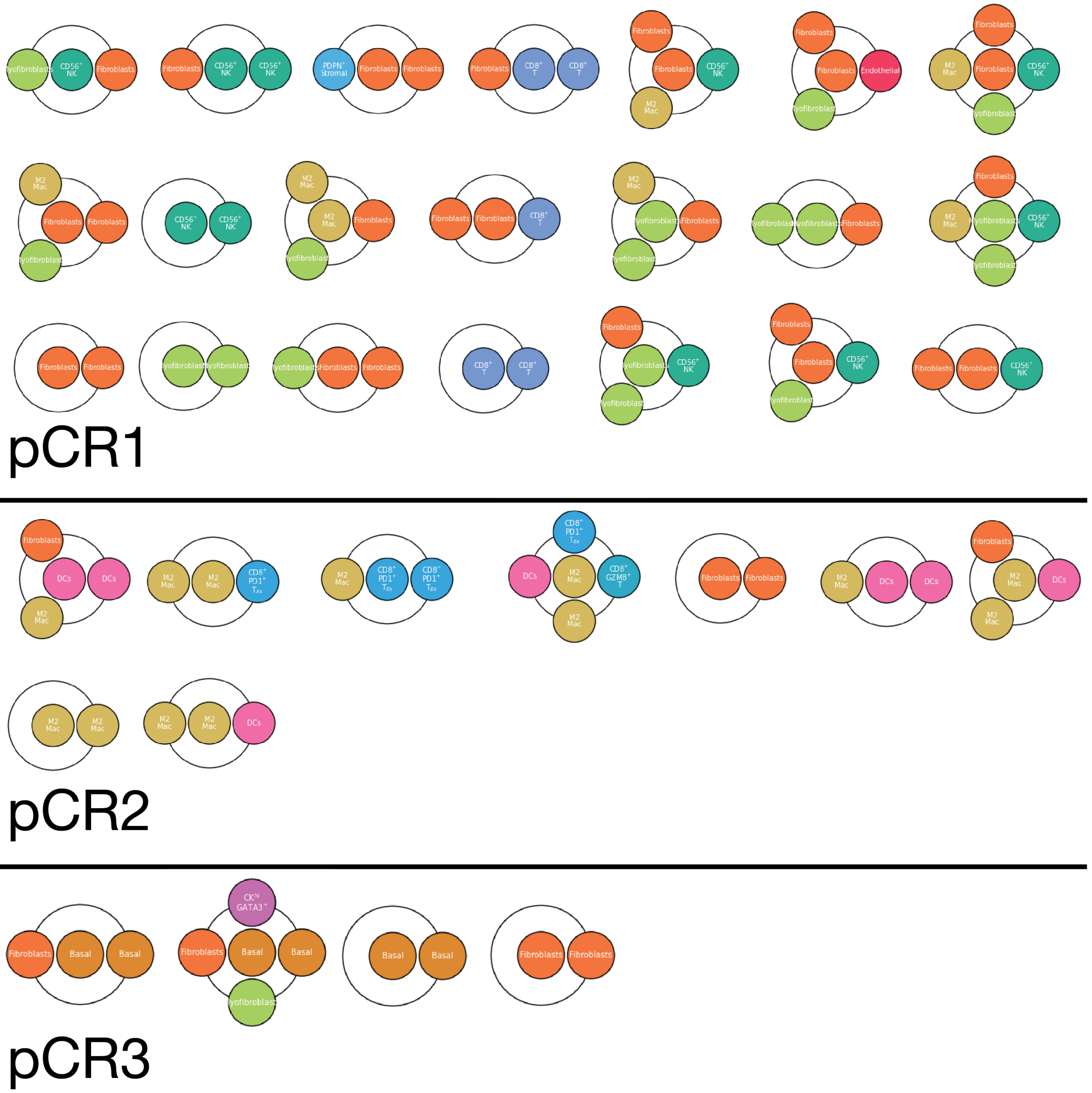

Supplementary Figure 8: List of enriched pathological complete response (pCR) N-Orbits from the pCR tissue cellular neighborhood (TCN) hotspots from the Wang *et al*. TNBC dataset.

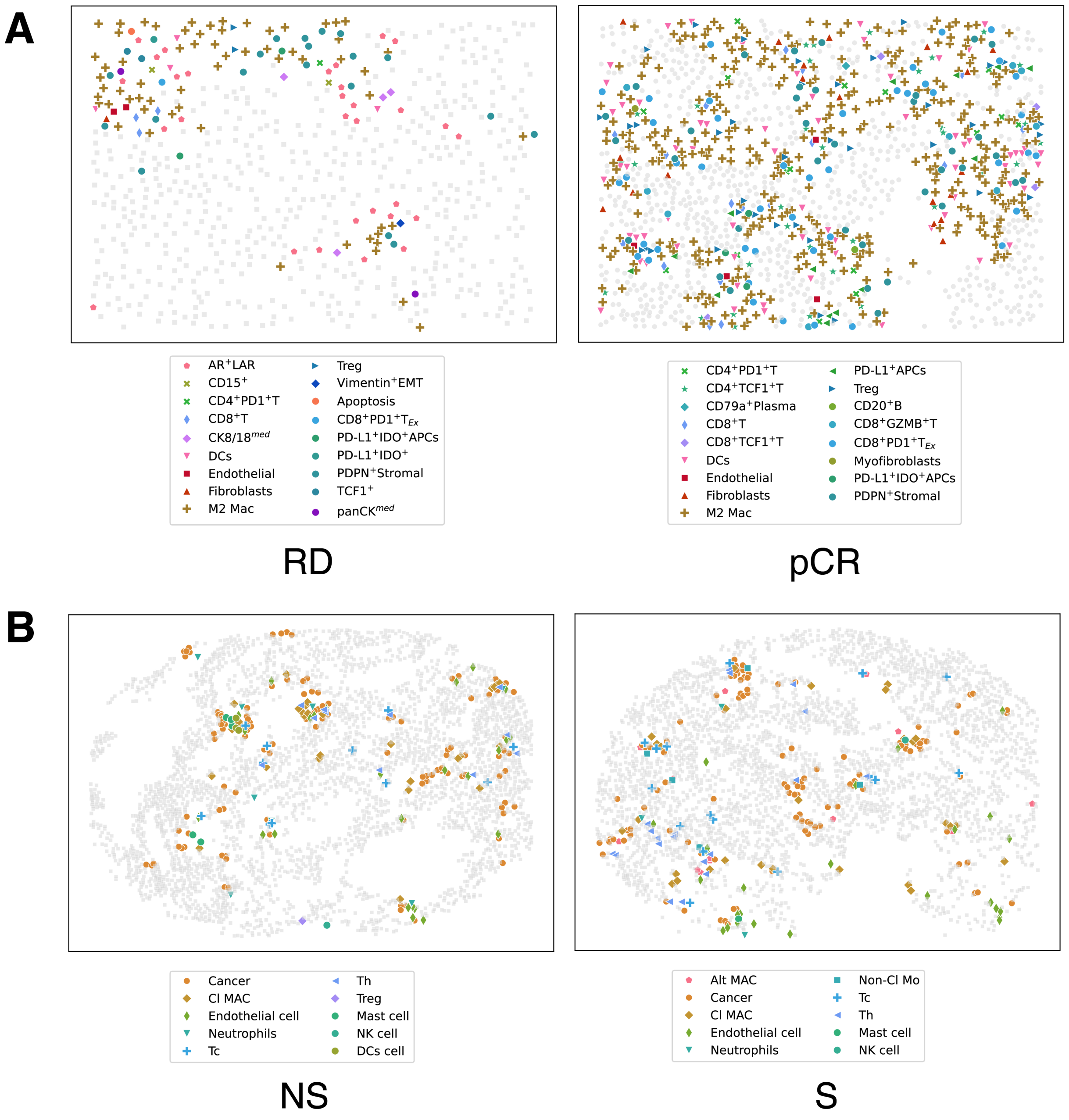

Supplementary Figure 9: Additional examples for the triple-negative breast cancer (TNBC) and non-small cell lung cancer (NSCLC) datasets by outcome. A) Residual disease (RD) and pathological complete response (pCR) TCN examples with similar cell type composition (Pearson R = 0.86) but varying spatial relationships (N-Orbit distance = 5.04) from the TNBC dataset. Left, positions on the UMAP representation of N-Orbit TCN distance; Right, TCN cell type maps. Grey squares represent cells outside the TCN. **B)** NS and S TCN examples with similar cell type composition (Pearson R = 0.995) but varying spatial relationships (N-Orbit distance = 1.82) from the NSCLC dataset. Left, positions on the UMAP representation of N-Orbit TCN distance. Right, TCN cell type maps. Grey squares represent cells outside the TCN.

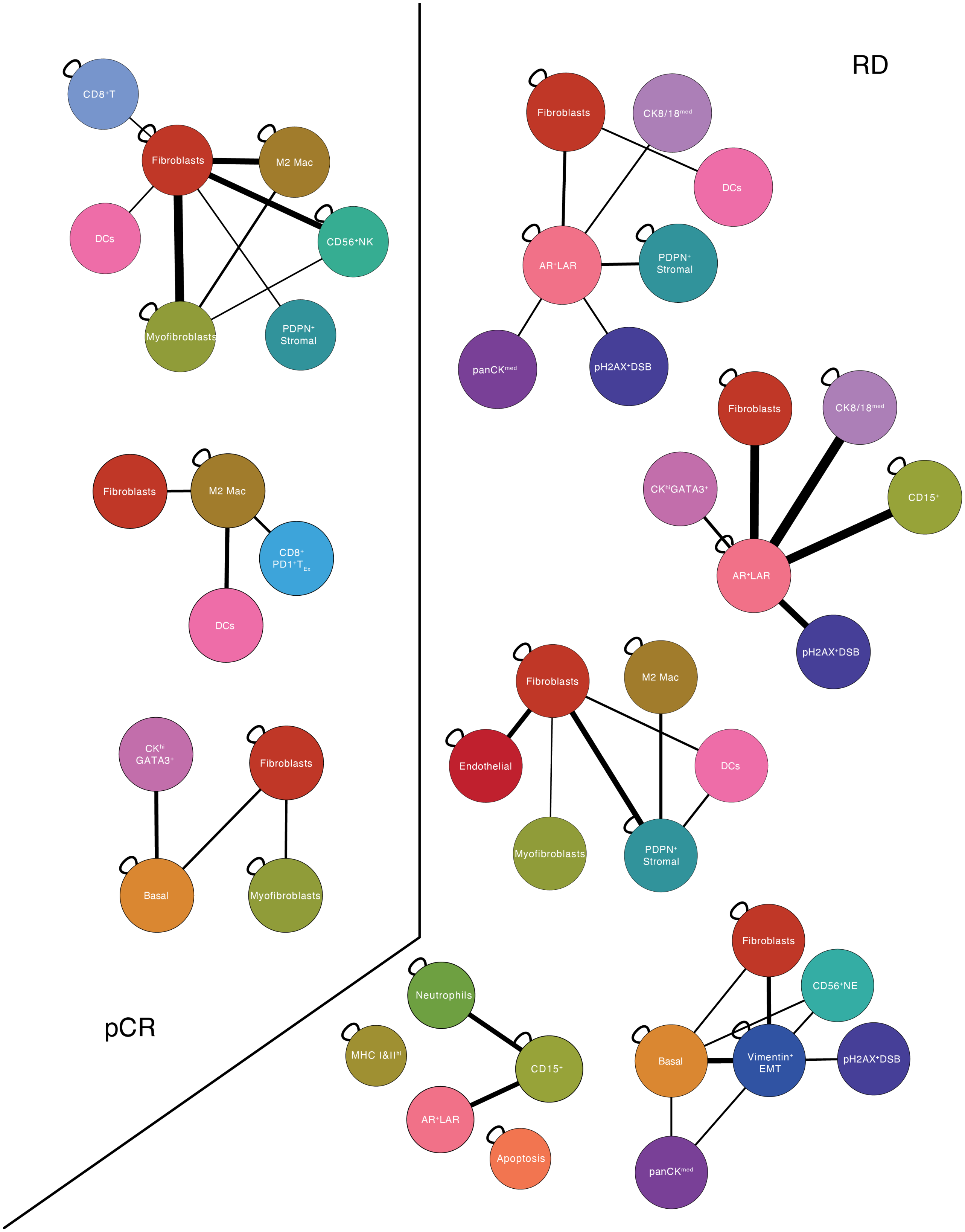

Supplementary Figure 10: Summary graphs from using CellCharter as the tissue cellular neighborhood (TCN) detection method on the triple-negative breast cancer (TNBC) dataset.

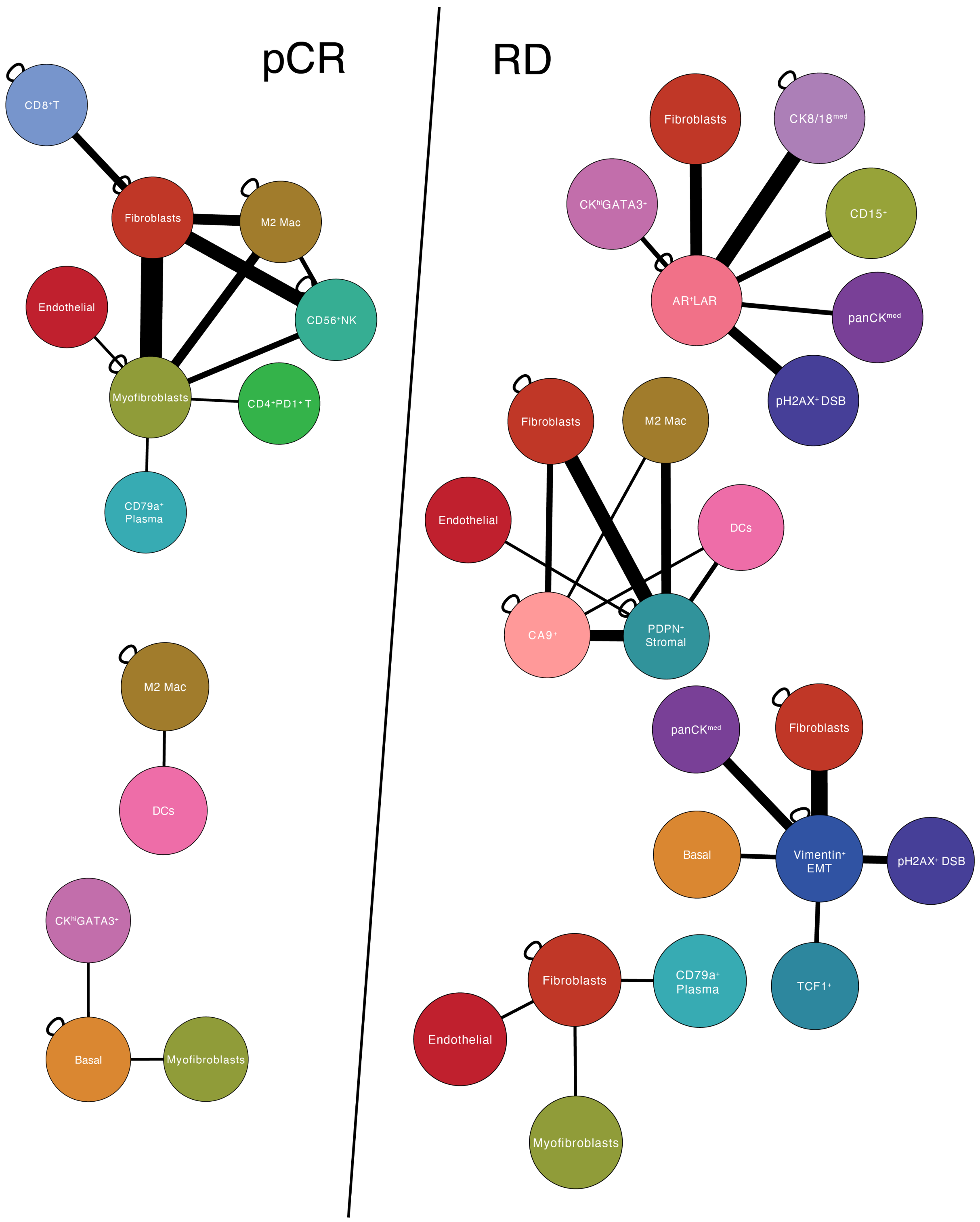

Supplementary Figure 11: Summary graphs from using BANKSY as the tissue cellular neighborhood (TCN) detection method on the triple-negative breast cancer (TNBC) dataset.

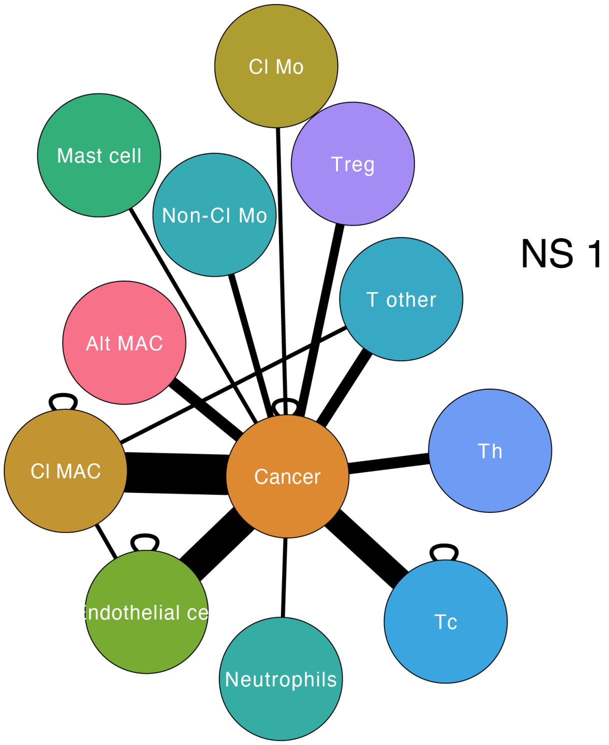

Supplementary Figure 12: Full summary graph for tissue cellular neighborhood (TCN) hotspot from the Sorin *et al*. non-small cell lung cancer (NSCLC) dataset that was pruned in the main figure (NS1). Edge weights indicate the relative recurrence of cell type co-memberships among enriched N-Orbits. Self-edges indicate monotypic nucleus-orbit relationships of that cell type. Cl MAC, CD163- macrophage; Alt MAC, CD163+ macrophage; Cl Mo, classical monocyte; Non-Cl Mo, non-classical monocyte; Int Mo, intermediate monocyte; Tc, cytotoxic T cell; Th, helper T cell; Treg, regulatory T cell.

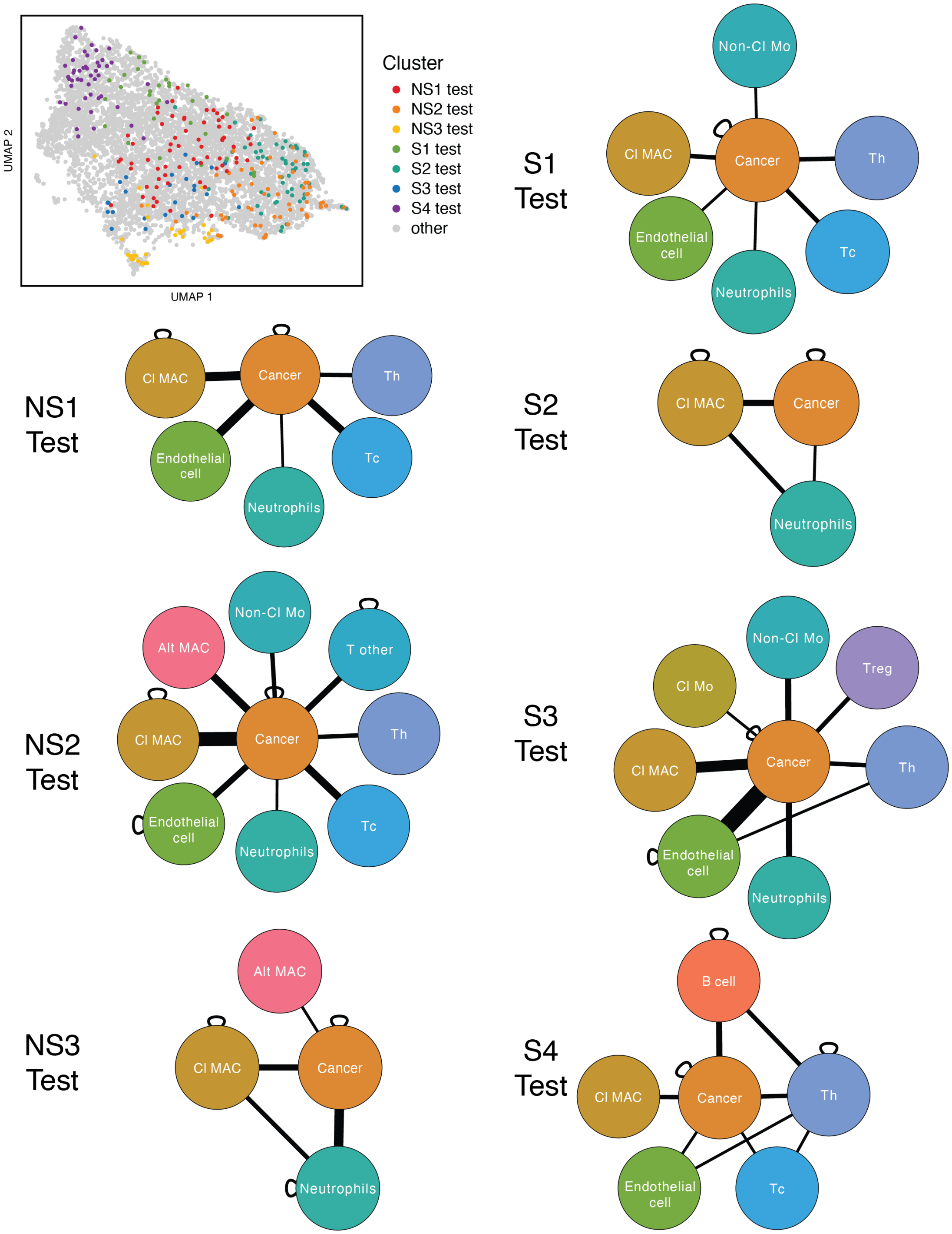

Supplementary Figure 13: Test set summary graphs for tissue cellular neighborhood (TCN) hotspots from the Sorin *et al.* NSCLC dataset, with accompanying TCN UMAP. A) Survival (S) and non-survival (NS) TCN hotspots plotted on the UMAP of the TCN distance matrix. TCN outside hotspots were plotted in gray for reference. **B-C**) Summary graphs. Source data are provided as a Source Data file.

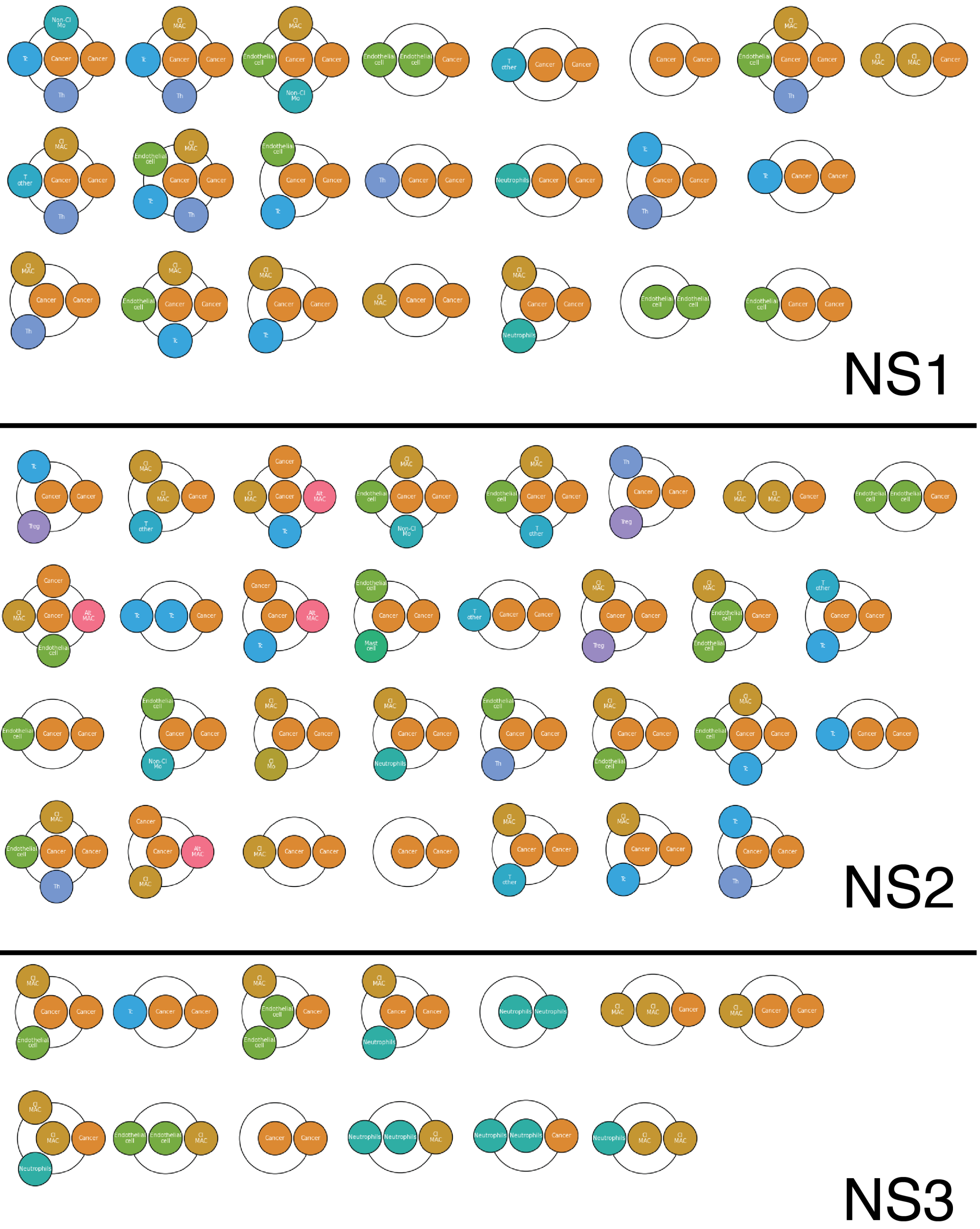

Supplementary Figure 14: List of enriched N-Orbits from the NS TCN clusters from the Sorin *et al*. NSCLC dataset.

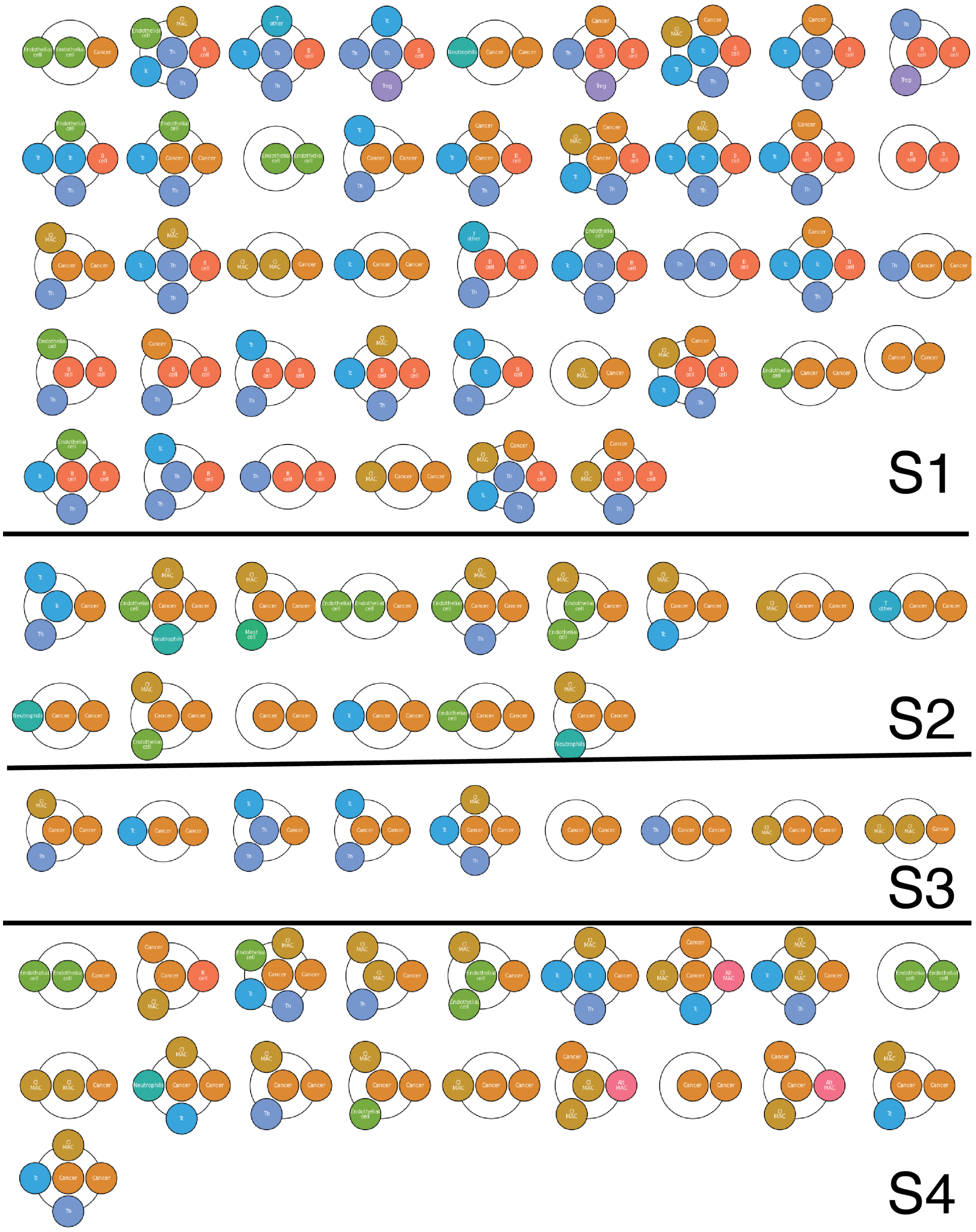

Supplementary Figure 15: List of enriched N-Orbits from the S TCN clusters from the Sorin *et al*. NSCLC dataset.

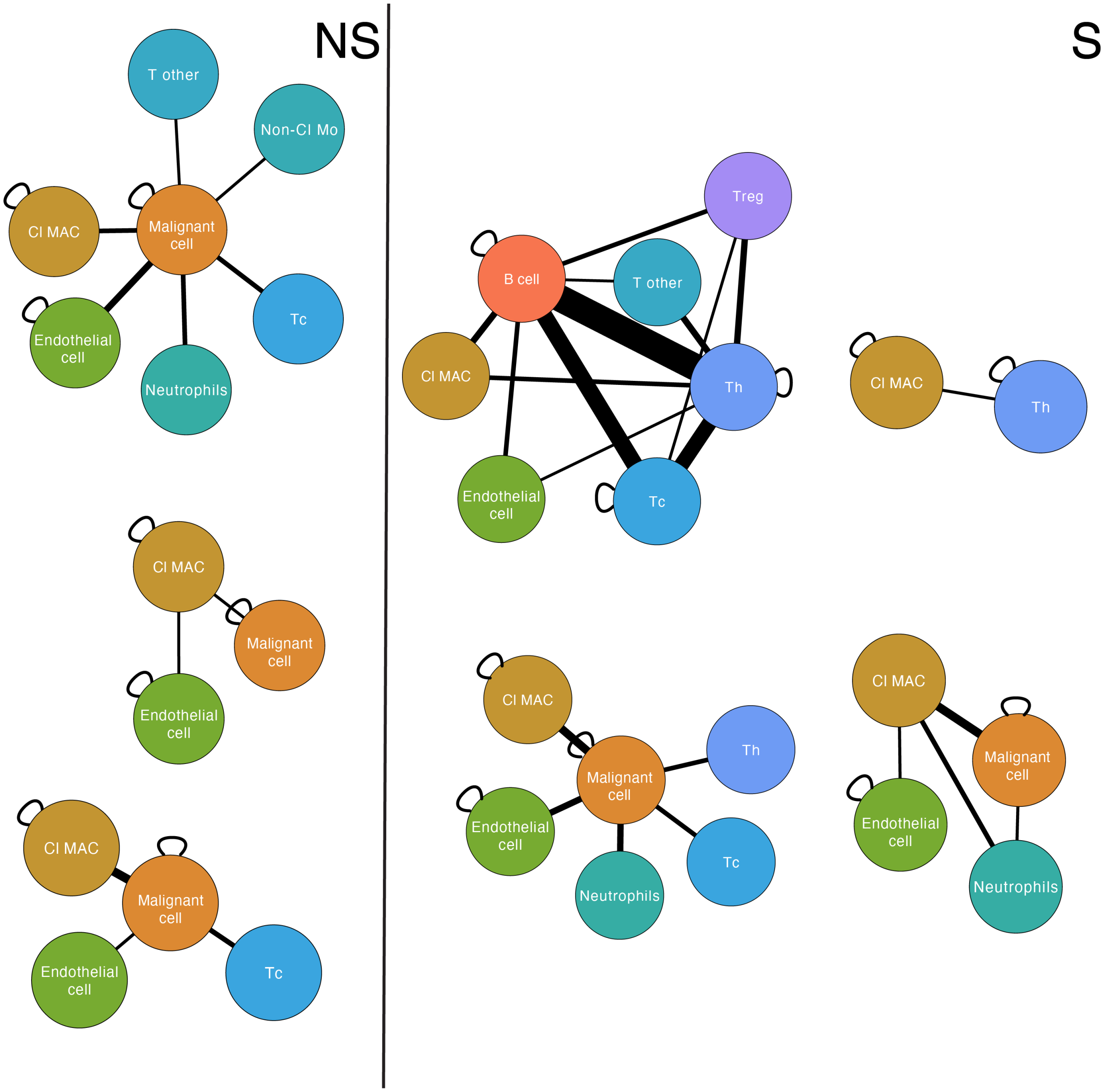

Supplementary Figure 16: Summary graphs from using CellCharter as the TCN detection method on the NSCLC dataset.

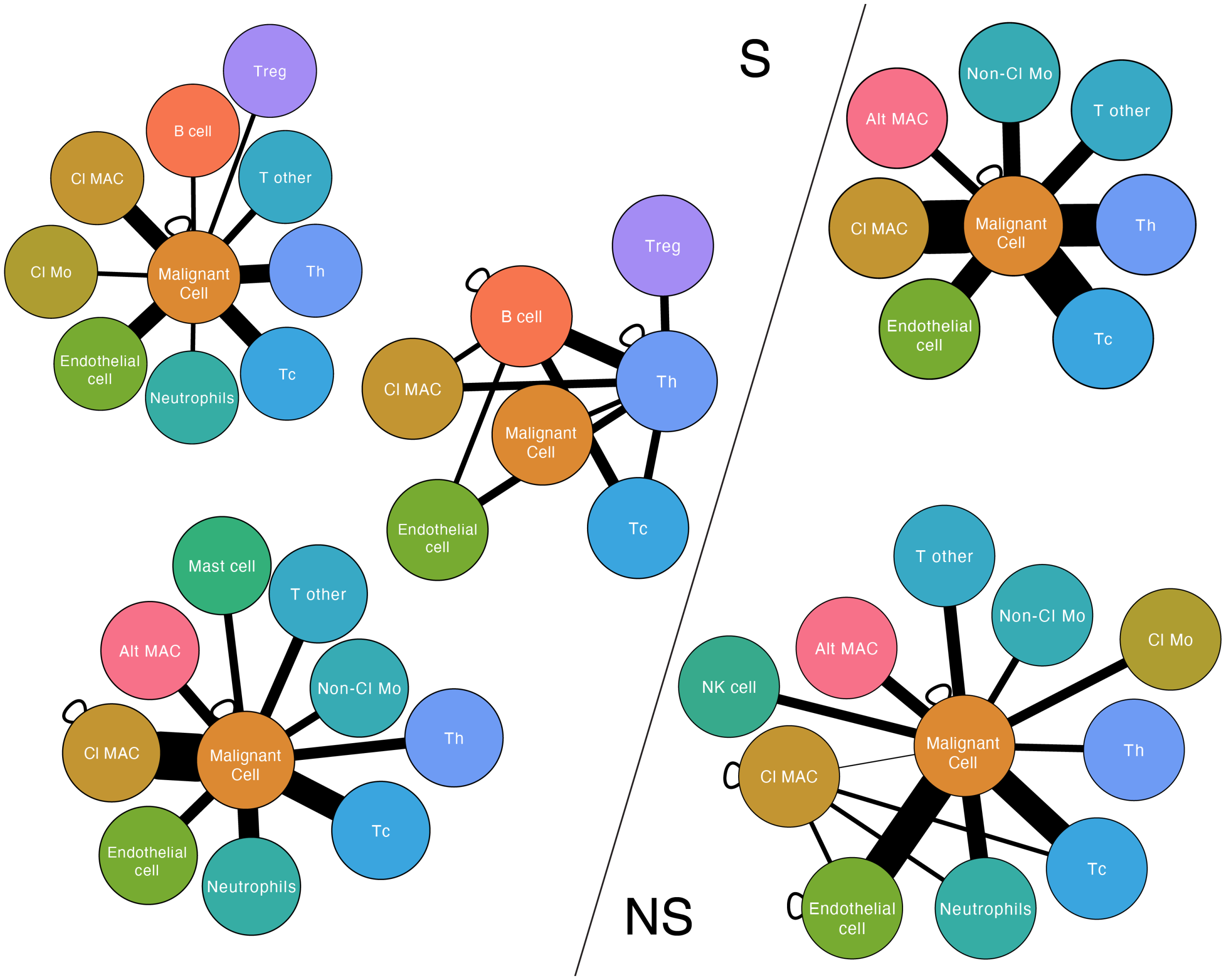

Supplementary Figure 17: Summary graphs from using BANKSY as the TCN detection method on the NSCLC dataset

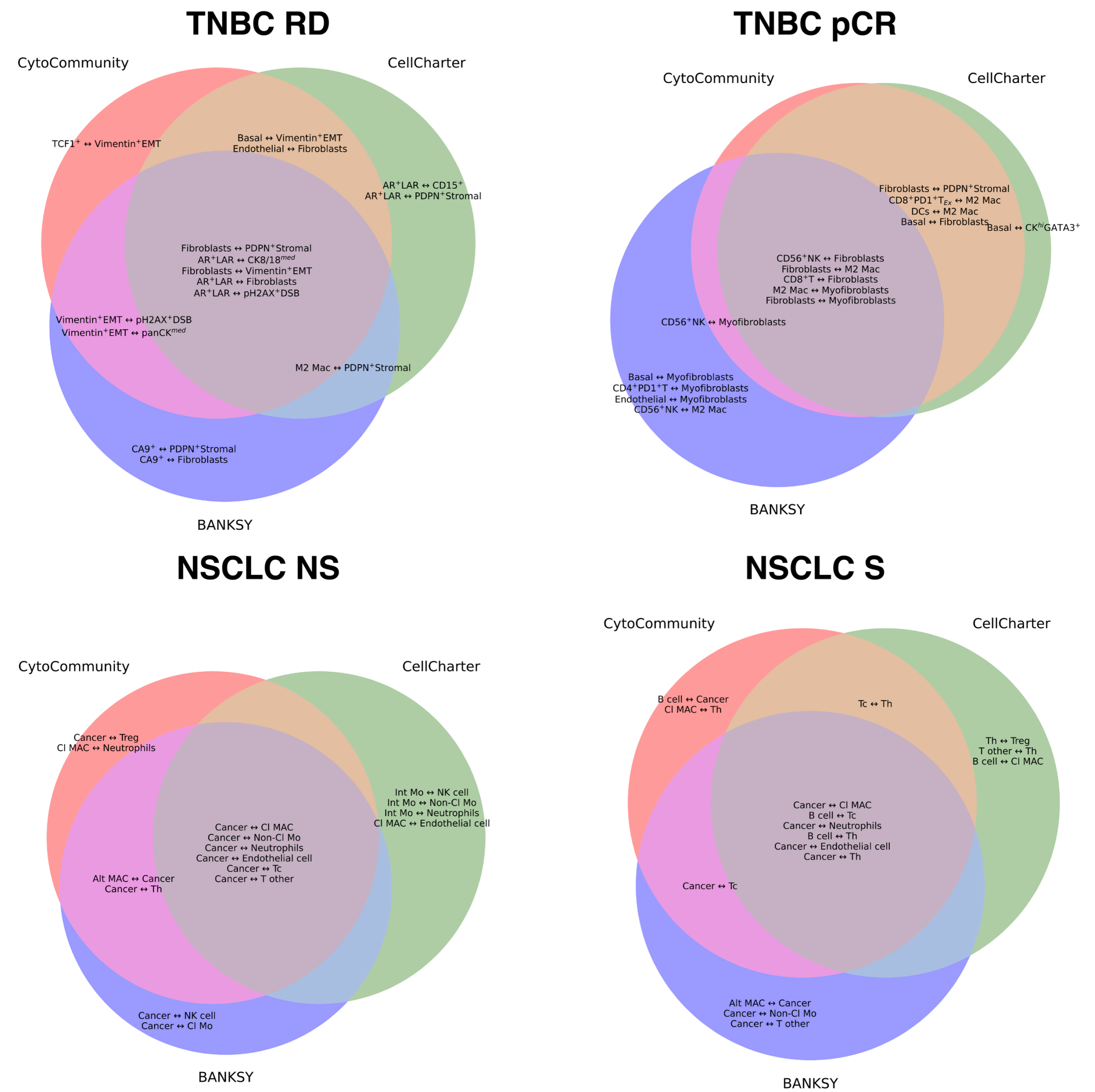

Supplementary Figure 18: Venn diagram of top 10 cell type pairs in clinical outcome hotspots by TCN caller for TNBC and NSCLC datasets. A) TNBC RD group. **B**) TNBC pCR group. **C**) NSCLC NS group. **D**) NSCLC S group.

Dataset	Fig	Authors	Source Publication Title	Link
CODEX Mouse Spleen	3	Goltsev et al.	Deep Profiling of Mouse Splenic Architecture with CODEX Multiplexed Imaging	https://doi.org/10.1016/j.cell.2018.07.010
MERFISH Mouse Hypothalamus	3	Moffitt et al.	Molecular, Spatial and Functional Single-Cell Profiling of the Hypothalamic Preoptic Region	https://doi.org/10.1126/science.aau5324
Xenium Pulmonary Fibrosis	3	Vannan et al.	Spatial transcriptomics identifies molecular niche dysregulation associated with distal lung remodeling in pulmonary fibrosis	https://doi.org/10.1016/j.cell.2024.03.013
TNBC Chemo +/- Immunotherapy	4	Wang et al.	Spatial predictors of immunotherapy response in triple-negative breast cancer	https://doi.org/10.1038/s41588-025-02080-x
NSCLC	5	Soren et al.	Single-cell spatial landscapes of the lung tumour immune microenvironment	https://doi.org/10.1038/s41586-022-05672-3
Mouse Brain Aging	6	Allen et al.	Molecular and spatial signatures of mouse brain aging at single-cell resolution	https://doi.org/10.1016/j.cell.2022.12.010
Macaque Cortex	6	Chen et al.	Single-cell spatial transcriptome reveals cell-type organization in the macaque cortex	https://doi.org/10.1016/j.cell.2023.06.009

Supplementary Table 1: List of datasets analyzed in this manuscript.

Dataset	Comparison	Neighborhood Detection	Method	AUROC	DeLong p-value (one-sided)
CODEX Spleen	Compartment Instance	Ground Truth	N-Orbit	0.941	
CODEX Spleen	Compartment Instance	Ground Truth	CTE	0.729	3.73E-25
CODEX Spleen	Compartment Instance	Ground Truth	BANKSY	0.747	
CODEX Spleen	Compartment Instance	Ground Truth	CellCharter	0.708	
CODEX Spleen	Compartment Instance	Ground Truth	SpaGCN	0.623	
CODEX Spleen	Compartment Instance	Ground Truth	GraphST	0.679	
MERFISH Hypothalamus	Hypothalamic Nuclei (R/L)	Ground Truth	N-Orbit	0.749	
MERFISH Hypothalamus	Hypothalamic Nuclei (R/L)	Ground Truth	CTE	0.617	3.45-9
MERFISH Hypothalamus	Hypothalamic Nuclei (R/L)	Ground Truth	BANKSY	0.689	
MERFISH Hypothalamus	Hypothalamic Nuclei (R/L)	Ground Truth	CellCharter	0.691	
MERFISH Hypothalamus	Hypothalamic Nuclei (R/L)	Ground Truth	SpaGCN	0.551	
MERFISH Hypothalamus	Hypothalamic Nuclei (R/L)	Ground Truth	GraphST	0.612	
Xenium PF	Histopathological Structure	Ground Truth	N-Orbit	0.843	
Xenium PF	Histopathological Structure	Ground Truth	CTE	0.621	9.76E-161
Xenium PF	Histopathological Structure	Ground Truth	BANKSY	0.661	
Xenium PF	Histopathological Structure	Ground Truth	CellCharter	0.78	
SyntheticV1	SMDJ vs. DJ (All)	Ground Truth	N-Orbit	1	
SyntheticV1	SMDJ vs. DJ (All)	Ground Truth	CTE	9.99E-01	1.00E+00
SyntheticV1	SMDJ vs. SJ (All)	Ground Truth	N-Orbit	0.893	
SyntheticV1	SMDJ vs. SJ (All)	Ground Truth	CTE	0.533	0.00E+00
SyntheticV1	SMDJ vs. SJ (A)	Ground Truth	N-Orbit	0.961	
SyntheticV1	SMDJ vs. SJ (A)	Ground Truth	CTE	0.516	0.00E+00
SyntheticV1	SMDJ vs. SJ (B)	Ground Truth	N-Orbit	0.989	
SyntheticV1	SMDJ vs. SJ (B)	Ground Truth	CTE	0.568	0.00E+00
SyntheticV1	SMDJ vs. SJ (C)	Ground Truth	N-Orbit	0.703	
SyntheticV1	SMDJ vs. SJ (C)	Ground Truth	CTE	0.516	3.05E-64
SyntheticV2	SMDJ vs. SJ	Ground Truth	N-Orbit	0.773	
SyntheticV2	SMDJ vs. SJ	Ground Truth	CTE	0.555	0.00E+00

Supplementary Table 2: List of one-sided DeLong p-values and AUROC values from benchmarking experiments described in Figures 1-3.

Dataset	Comparison	Neighborhood Detection	Method	p-value (One-Sided T-Test)
CODEX Spleen	Compartment Instance	Ground Truth	N-Orbit	3.42E-171
CODEX Spleen	Compartment Instance	Ground Truth	CTE	2.41E-24
CODEX Spleen	Compartment Instance	Ground Truth	BANKSY	0.00E+00
CODEX Spleen	Compartment Instance	Ground Truth	CellCharter	0.00E+00
CODEX Spleen	Compartment Instance	Ground Truth	SpaGCN	1.93E-124
CODEX Spleen	Compartment Instance	Ground Truth	GraphST	0.00E+00
MERFISH Hypothalamus	Hypothalamic Nuclei (R/L)	Ground Truth	N-Orbit	8.11E-31
MERFISH Hypothalamus	Hypothalamic Nuclei (R/L)	Ground Truth	CTE	2.30E-06
MERFISH Hypothalamus	Hypothalamic Nuclei (R/L)	Ground Truth	BANKSY	0.00E+00
MERFISH Hypothalamus	Hypothalamic Nuclei (R/L)	Ground Truth	CellCharter	0.00E+00
MERFISH Hypothalamus	Hypothalamic Nuclei (R/L)	Ground Truth	SpaGCN	0.00E+00
MERFISH Hypothalamus	Hypothalamic Nuclei (R/L)	Ground Truth	GraphST	0.00E+00
Xenium PF	Histopathological Structure	Ground Truth	N-Orbit	0.00E+00
Xenium PF	Histopathological Structure	Ground Truth	CTE	1.73E-47
Xenium PF	Histopathological Structure	Ground Truth	BANKSY	0.00E+00
Xenium PF	Histopathological Structure	Ground Truth	CellCharter	0.00E+00
SyntheticV1	SMDJ vs. DJ (All)	Ground Truth	N-Orbit	0.00E+00
SyntheticV1	SMDJ vs. DJ (All)	Ground Truth	CTE	0.00E+00
SyntheticV1	SMDJ vs. SJ (All)	Ground Truth	N-Orbit	0.00E+00
SyntheticV1	SMDJ vs. SJ (All)	Ground Truth	CTE	3.34E-09
SyntheticV1	SMDJ vs. SJ (A)	Ground Truth	N-Orbit	0.00E+00
SyntheticV1	SMDJ vs. SJ (A)	Ground Truth	CTE	1.68E-01
SyntheticV1	SMDJ vs. SJ (B)	Ground Truth	N-Orbit	0.00E+00
SyntheticV1	SMDJ vs. SJ (B)	Ground Truth	CTE	7.60E-13
SyntheticV1	SMDJ vs. SJ (C)	Ground Truth	N-Orbit	3.16E-124
SyntheticV1	SMDJ vs. SJ (C)	Ground Truth	CTE	4.80E-01
SyntheticV2	SMDJ vs. SJ	Ground Truth	N-Orbit	0.00E+00
SyntheticV2	SMDJ vs. SJ	Ground Truth	CTE	8.00E-45

Supplementary Table 3: List of p-values from one-sided t-tests from benchmarking experiments described in Figures 1-3.

Analysis	N-Orbit radius (r)	Nucleus change penalty (p)	N-Orbit bootstrap sample size (s)
SyntheticV1	100	1	1000
SyntheticV1 (Sample-Level)	100	1	1000
SyntheticV2	100	1	1000
SyntheticV2 (Sample-Level)	100	1	2000
CODEX Mouse Spleen (Instance)	100	1	5000
MERFISH Mouse Hypothalamus	100	1	1000
Xenium Pulmonary Fibrosis	100	5	1000
TNBC Chemo +/- Immunotherapy	100	1	1000
NSCLC	100	1	1000
Cortex Homology	100	1	5000

Supplementary Table 4: List of parameters used for N-Orbit distance calculation.
