# Supplementary material for: N-Orbit: towards a universal model and metric for comparing tissue microenvironments
Source: Nat Commun. 2026 May 22;17:6741. doi: 10.1038/s41467-026-73561-8 (PMC13385583; doi:10.1038/s41467-026-73561-8)
Supplement: Supplementary file 3 — Transparent Peer Review file [file 41467_2026_73561_MOESM3_ESM.pdf]

nature portfolio

# Peer Review File

# N-Orbit: towards a universal model and metric for comparing tissue microenvironments

Corresponding Author: Professor Kai Tan

This manuscript has been previously reviewed at another journal. This document only contains information relating to versions considered at Nature Communications.

**This file contains all reviewer reports in order by version, followed by all author rebuttals in order by version.**

Version 0:

Reviewer comments:

Reviewer #4

(Remarks to the Author)
The authors introduce N-Orbit, a conceptually novel and mathematically elegant framework for quantifying and interpreting differences among pre-defined Tissue Cellular Neighborhoods (TCNs) in spatial transcriptomics. I find the theoretical foundation of this work particularly compelling; it addresses a significant gap in the field—namely, the lack of a principled, alignment-free metric to compare tissue architectures across diverse biological contexts. The in-silico benchmarks (Figure 2) convincingly establish its theoretical merit in capturing spatial joint distributions beyond mere cell-type composition

However, while the mathematical "bones" of the algorithm are strong, the "flesh" of its biological applications (Figures 4–6) currently lacks the depth required to demonstrate N-Orbit's unique value to the broader spatial omics community. At present, the manuscript reads more like a technical proof-of-concept than a robust biological discovery tool. To increase the impact and "attractiveness" of this method for general users, more rigorous benchmarking against existing baselines and a clearer demonstration of its unique insights are essential. I recommend the authors to address the following concerns, as I believe the framework has the potential to become a standard reference for the field.

Technical / benchmark aspect:
1. As mentioned in Review #2 (regarding the design of the N-orbit), only considered the distance information of the nearest cell for each cell type to the “nucleus”, but has ignored factors such as the number or density of cells for each cell type within the neighborhood. Such a design decision needs to be proven to be reasonable, e.g. through ablation test.

2. Reviewer #3’s misapprehension that N-Orbit itself defines TCNs underscores a broader concern: the manuscript is frequently too terse, inviting confusion. Please expand the Methods and figure legends to spell out—preferably with a workflow cartoon—that N-Orbit is strictly a post-hoc, alignment-free distance metric computed on neighborhoods already delineated by an upstream caller, and add a dedicated Discussion subsection that explicitly lists the method’s limitations: it cannot detect neighborhoods de novo, its biological interpretability hinges mainly on the accuracy of those upstream TCNs and their cell-type annotations, and other limitations. Clarifying these points will pre-empt similar misunderstandings and give readers an unambiguous picture of what N-Orbit can and cannot deliver.

3. Because N-Orbit itself does not delineate TCNs, its downstream interpretations are contingent on the upstream algorithm that does. The authors must therefore benchmark the sensitivity of their results to the choice of TCN-calling method in their application scenarios (Figures 4-6). Repeating key analyses with alternative, independently published TCN pipelines and quantifying congruence will clarify whether the reported associations are robust or merely an artefact of a single TCN definition.

4. For the hotspots identified in Figures 4–6, the authors need to repeat the identical Louvain-clustering and enrichment workflow using the embedding distance matrix produced by the same algorithm and other algorithms that generated TCNs. Only if N-Orbit uniquely recovers the treatment-response or survival-associated neighborhoods can its added value be convincingly claimed.

5. The manuscript never evaluates downstream predictive performance when the embeddings produced by the upstream TCNs algorithms are supplied to the classifier. Without this baseline, it could not assert that N-Orbit distances are the superior feature set.

Biological applications aspect:
6. To help readers understand and translate the N-Orbit framework into their own studies, I strongly encourage the authors to select 1~2 representative examples from Figures 4-6, draw a concise schematic plot to show how to use the tool for downstream analysis, and expand the corresponding Results text to a full paragraph that includes detailed information.

7. Take Figure 4 as an example, the following scenario needs to be presented: one TCN pair that possesses virtually identical cell-type composition (e.g., 40 % $CD8^{+}$ T, 30 % fibroblast, 30 % tumour) but occupies opposite extremes of the N-Orbit distance space (one pCR-hotspot, one RD-hotspot). Provide side-by-side zoom-in FOVs (could refer to the CytoCommunity Fig. 5d-h, https://www.nature.com/articles/s41592-023-02124-2#Fig5), their N-Orbit vector heat-maps, and the resulting pairwise distances, then conclude with a one-sentence takeaway—"same composition, different architecture, opposite clinical outcome." Such a contrast will make the added value of N-Orbit instantly intuitive to a broad readership.

8. Guidance for Reuse: the concept is appealing, but the application examples in Figures 4-6 are still confusing when read in isolation and give no blueprint for reuse. I encourage the authors to further explore the mechanism of their application scenario examples and discuss what kind of scenarios are suitable for applying this method. I also suggest the authors to add a dedicated discussion subsection listing the method's limitations

Minor Point:
1. The term "interaction" in the manuscript does not denote a ligand–receptor pair or a spatial co-localization coefficient; instead, it is quantified by the edge weight in the N-Orbit summary graph, which simply records how frequently two cell types co-occur within the same N-Orbit. The authors should either demonstrate that this frequency metric genuinely reflects biologically meaningful cell–cell interactions or adopt a more precise term to avoid conceptual overstatement.

2. As mentioned in Review #3 (regarding the concept TCN, neighborhood), the manuscript alternates between "TCNs" and "neighborhoods" without stating whether they denote the same entity. Please provide a single, explicit definition and thereafter use one term consistently; if a distinction is intended, clarify this up-front and avoid interchangeable usage.

3. Superscripts in the manuscript and figures are typed as "^" rather than formatted text (e.g., "CD15^+"). Please replace all ASCII carets with true superscript characters throughout the main text, figure panels, and legends to meet Nature Communications production standards.

(Remarks on code availability)

Version 1:

Reviewer comments:

Reviewer #4

(Remarks to the Author)
The authors have provided a thorough and constructive response to the initial reviewer comments. I commend the improvements made, particularly the clarification of the method's positioning within the spatial omics workflow, the revised terminology that avoids biological overinterpretation, and the inclusion of helpful schematic diagrams. These revisions significantly enhance the clarity of the manuscript. However, while the technical performance benchmarks have been strengthened, several key concerns regarding the biological interpretability and practical usability of N-Orbit remain to be fully addressed.

Technical / benchmark evaluation:

1. (For previous Q1) The authors provide a reasonable ablation test comparing N-Orbit to a density-based alternative (Supplementary Figure 3G). However, the description lacks critical methodological details that prevent full assessment of this comparison. Furthermore, the hypothalamus results in Figure S3G imply that the density-based approach occasionally outperforms N-Orbit, raising the question of whether integrating density information into N-Orbit could expand the method's applicability.

2. (For previous Q3 and Q4) While the authors have shown that N-Orbit consistently outperforms CTE across several upstream TCN callers (e.g., BANKSY, CellCharter), the rebuttal still lacks a rigorous quantitative assessment of the variance introduced by these different inputs. It is insufficient to merely state that results are 'similar' or that N-Orbit remains superior to a baseline.
To justify N-Orbit as a robust, alignment-free metric, the authors must provide a detailed benchmarking of the cross-method stability. Specifically:
(1) Please quantify the degree of divergence in N-Orbit output features (e.g., pairwise distance matrices or embedding

distributions) when using different TCN callers. A simple comparison of AUROC is not enough; a more granular analysis, such as a concordance study of the resulting spatial embeddings, is required to measure how much the 'choice of caller' shifts the N-Orbit coordinate space.
(2) The authors should evaluate whether the performance gain of N-Orbit is sensitive to the granularity or the specific hyper-parameters of the upstream TCN detection. This will clarify if the metric's performance is truly intrinsic to N-Orbit or heavily buffered by the quality of the specific upstream tool used.

Biological applications aspect:
3. (For previous Q3, Q4, and Q8) Beyond technical benchmarks, my primary concern remains the convergence of biological discoveries. A universal metric should ideally lead to the same biological interpretation regardless of the upstream preprocessing, yet this 'universality' has not been sufficiently demonstrated.
If the downstream biological findings—such as specific cell-type co-occurrence patterns, spatial domain signatures, or patient clinical stratifications—shift significantly depending on the TCN caller, the utility of N-Orbit as a plug-and-play metric is limited.
To address this, please (1) demonstrate whether the top-ranked biological features or hotspots identified in the TNBC dataset are preserved across different TCN frameworks. (2) In light of the observed input-dependency, the authors must provide a principled guideline for selecting upstream TCN methods. For instance, in which tissue contexts (e.g., highly structured vs. diffuse) is a graph-based caller (like CellCharter) preferred over a spatial-neighbor-based one (like BANKSY) when using N-Orbit? Why? (3) Please include a dedicated discussion on the performance divergency from upstream callers to N-Orbit. Users need to understand the expected variance in biological output and the potential risks of unreal discoveries if a suboptimal TCN framework is chosen for a specific biological system.

(Remarks on code availability)
Remarks on code availability:
4. Additionally, I have tested the N-Orbit codebase available on GitHub. While the core algorithm is functional, the output matrices lack necessary row and column labels, rendering them difficult for users to interpret or integrate into downstream pipelines. Furthermore, the lack of clear documentation on output interpretation hinders the utility of the tool.
To improve reproducibility and community accessibility, I strongly recommend that the authors: (1) Enhance code documentation: Include clear row/column headers in all output matrices and provide a detailed explanation of the output format in the documentation. (2) Provide a demo pipeline: Include a representative, ready-to-run sample dataset (e.g., a small subset of the TNBC or NSCLC data) with a corresponding tutorial (script or notebook). This will allow users to verify their installation and understand the expected input/output structure.

Version 2:

Reviewer comments:

Reviewer #4

(Remarks to the Author)
The authors have provided a comprehensive and constructive rebuttal that addresses the technical and biological concerns raised in the previous review. The revised manuscript significantly strengthens the evidence for N-Orbit as a robust, alignment-free metric for comparing tissue cellular neighborhoods (TCNs). By providing granular quantitative assessments and clear methodological guidelines, the authors have demonstrated the utility and stability of the framework. I recommend the paper for publication in Nature Communications.

(Remarks on code availability)
They added more detail in the README and added an example synthetic dataset in this revision. These additions improve community accessibility.

**Open Access** This Peer Review File is licensed under a Creative Commons Attribution 4.0 International License, which permits use, sharing, adaptation, distribution and reproduction in any medium or format, as long as you give appropriate credit to the original author(s) and the source, provide a link to the Creative Commons license, and indicate if changes were made.
In cases where reviewers are anonymous, credit should be given to 'Anonymous Referee' and the source.
The images or other third party material in this Peer Review File are included in the article's Creative Commons license, unless indicated otherwise in a credit line to the material. If material is not included in the article's Creative Commons license and your intended use is not permitted by statutory regulation or exceeds the permitted use, you will need to obtain permission directly from the copyright holder.
To view a copy of this license, visit https://creativecommons.org/licenses/by/4.0/

**Point-by-point response to the reviewer's comments**

***Technical / benchmark aspect***

1. As mentioned in Review #2 (regarding the design of the N-orbit), only considered the distance information of the nearest cell for each cell type to the "nucleus", but has ignored factors such as the number or density of cells for each cell type within the neighborhood. Such a design decision needs to be proven to be reasonable, e.g. through ablation test.

The reviewer makes a valid point that N-Orbit does not explicitly capture cell type density within an individual vector. However, we assert that density is indirectly encoded in N-Orbit vectors, particularly when considering them in aggregate. In regions of higher local abundance of a given cell type, the expected nearest-neighbor distance of that cell type is consistently lower across the cells in that region. Conversely, sparsely distributed cell types will result in larger nearest-neighbor distances.

To demonstrate the superiority of a nearest-neighbor distance approach over a density-based one, we designed an alternative distance metric based on a vector formulation that encodes only the number of cells of each cell type within a specified radius (Supplementary Figure 3G). We compared this density-based metric with N-Orbit for the three manually-annotated datasets benchmarked in Figure 3 across the same search radii used for hyperparameter testing in Supplementary Figures 3A and 3C. We observed substantially superior performance of N-Orbit in most tests, as well as much lower sensitivity to the radius hyperparameter. Moreover, we found that N-Orbit asymptotically improved performance at higher radii, whereas the density-based approach generally decreased performance as the search scale increased. Taken together, we believe N-Orbit to be both superior in performance and robustness to scale compared to a density-based approach.

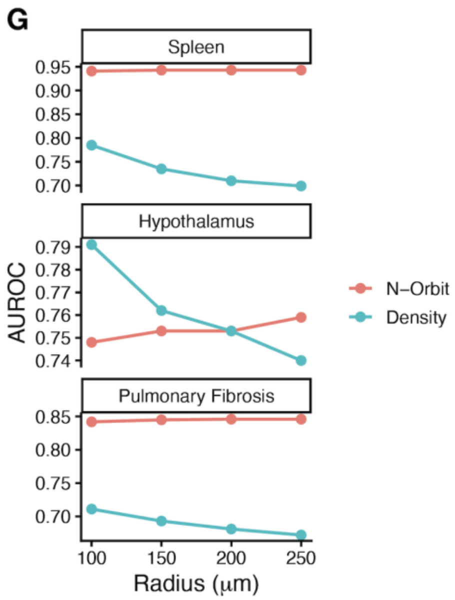


**Supplementary Figure 3G.** Comparison to a density-based alternative vector and distance formulation across various radii in the spleen, hypothalamus, and pulmonary fibrosis datasets.

2. Reviewer #3's misapprehension that N-Orbit itself defines TCNs underscores a broader concern: the manuscript is frequently too terse, inviting confusion. Please expand the Methods and figure legends to spell out—preferably with a workflow cartoon—that N-Orbit is strictly a post-hoc, alignment-free distance metric computed on neighborhoods already delineated by an upstream caller, and add a dedicated Discussion subsection that explicitly lists the method's limitations: it cannot detect neighborhoods de novo, its biological interpretability hinges mainly on the accuracy of those upstream TCNs and their cell-type annotations, and other limitations. Clarifying these points will pre-empt similar misunderstandings and give readers an unambiguous picture of what N-Orbit can and cannot deliver.

We appreciate the reviewer's comment, which helps clarify the purpose of N-Orbit and distinguish it from more conventionally known TCN detection methods. We have made three new additions to clarify this point: 1) A new panel A in Figure 1 to visually depict N-Orbit as separate from and downstream of existing TCN detection methods; 2) An "Inputs" subsection at the beginning of Methods clarifying its use as a *post hoc* alignment-free distance metric for TCNs generated by a separate detection method; and 3) a dedicated "Limitations" subsection as part of the Discussion, further reiterating that N-Orbit does not generate TCNs *de novo*. Altogether, we believe these changes should clear up any potential confusion for readers about the purpose and use of N-Orbit.

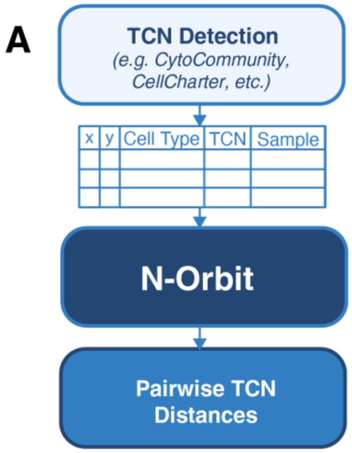


**Figure 1A.** Before performing N-Orbit, TCNs are assigned using a separate detection method, such as CytoCommunity or CellCharter. From these pre-specified TCNs, N-Orbit computes pairwise distances between them based on the spatial proximity of constituent cell types.

3. Because N-Orbit itself does not delineate TCNs, its downstream interpretations are contingent on the upstream algorithm that does. The authors must therefore benchmark the sensitivity of their results to the choice of TCN-calling method in their application scenarios (Figures 4-6). Repeating key analyses with alternative, independently published TCN pipelines and quantifying congruence will clarify whether the reported associations are robust or merely an artefact of a single TCN definition.

We agree with the reviewer that it is important to demonstrate a reasonable degree of consistency in performance across upstream TCN-calling methods, although some variation is expected due to differences in underlying TCN structures. We have

repeated our TNBC and NSCLC analyses using BANKSY and CellCharter (both used previously for benchmarking in Figure 3). We did not include SpaGCN and GraphST due to their poorer performance on the simpler benchmarking tasks in Figure 3 and their substantially slower runtime. However, we would also like to emphasize that N-Orbit could, in principle, be used to overcome the lack of multi-sample alignment in these methods to generate integrated embeddings.

Both BANKSY and CellCharter performed similarly on the TNBC and NSCLC datasets. Although there was a slight drop in performance from the original CytoCommunity-based model on the TNBC dataset, we found that BANKSY's superiority over cell type enrichment-based prediction held in all tested cases (Supplementary Figures 4A, 4D).

Because the analysis in Figure 6 was originally performed using manually annotated TCNs, we did not include the mouse and macaque brain datasets for this task (as well as those in Comments 5 and 6). Previously, TCN detection methods had difficulty reconstructing the cortical layers accurately (separately from the use of N-Orbit), so manual annotations were used instead.

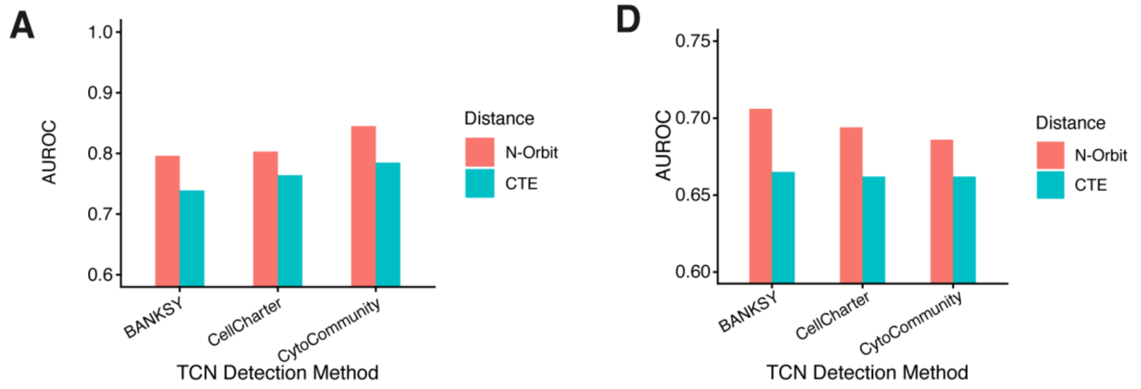


**Supplementary Figure 4.** Additional benchmarking for TNBC and NSCLC datasets. A) TNBC outcome prediction performance on TCNs generated using alternative methods. D) NSCLC outcome prediction performance on TCNs generated using alternative methods.

4. For the hotspots identified in Figures 4–6, the authors need to repeat the identical Louvain-clustering and enrichment workflow using the embedding distance matrix produced by the same algorithm and other algorithms that generated TCNs. Only if N-Orbit uniquely recovers the treatment-response or survival-associated neighborhoods can its added value be convincingly claimed.

Similar to comment #3, we agree with the reviewer that it is important to confirm consistency in qualitative insights across different upstream TCN-calling methods. Some variation is expected due to differences in underlying TCNs. Nevertheless, we observed similar themes in both TNBC and NSCLC datasets when using BANKSY and CellCharter compared to CytoCommunity (Supplementary Figures 10, 11, 16, 17). There was some variation in the grouping of summary graphs and minor connections between cell types. In aggregate, for example, RD summary graphs between CytoCommunity and CellCharter, were relatively consistent. To quantitatively assess

this, we calculated Spearman correlations between the maximal edge weights for all pairs of cell types in graphs aggregated by outcome group across TCN versions (Supplementary Figures 4C, 4F). These correlations were moderate to strong for all datasets, outcome groups, and TCN detection methods.

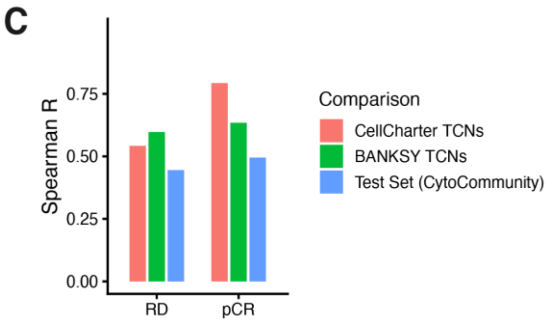


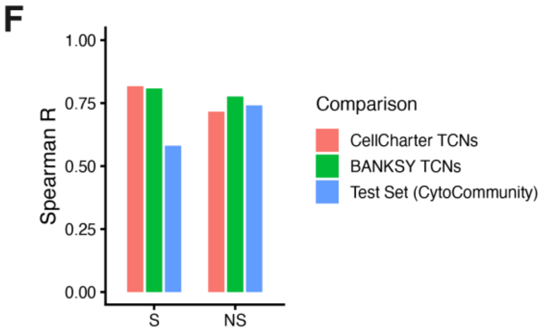


**Supplementary Figure 4. Additional benchmarking for TNBC and NSCLC datasets. C)** Spearman correlation between maximal RD and pCR summary graph edge weights using CellCharter (training set), BANKSY (training set), and CytoCommunity (test set), compared with CytoCommunity (training set) on the TNBC dataset. **F)** Spearman correlation between maximal S and NS summary graph weights using CellCharter (training set), BANKSY (training set), and CytoCommunity (test set) compared with CytoCommunity (training set) on the NSCLC dataset.

5. The manuscript never evaluates downstream predictive performance when the embeddings produced by the upstream TCNs algorithms are supplied to the classifier. Without this baseline, it could not assert that N-Orbit distances are the superior feature set.

We appreciate the reviewer's suggestion to also demonstrate superiority over alternative embeddings (from BANKSY and CellCharter) in addition to the CTE-only models. Since these embeddings are generated at the cell level rather than the TCN level, we aggregated them by averaging (i.e., taking the center of mass within the embedding space). When comparing N-Orbit against embeddings generated by the same method used to create the underlying TCNs, N-Orbit outperformed in 3 out of 4 cases. For the TNBC dataset using the CellCharter-generated TCNs, the CellCharter embeddings performed similarly to N-Orbit features. However, the best performance on this dataset was still achieved by N-Orbit features on the CytoCommunity-generated TCNs (Supplementary Figures 4B, 4E). Since CytoCommunity does not provide latent embeddings, such as BANKSY and CellCharter, a *post hoc* tool such as N-Orbit would be necessary to generate these features. This further emphasizes the utility of N-Orbit through its ability to enhance downstream analyses of TCN detection methods, which may otherwise have limitations inherent to their design.

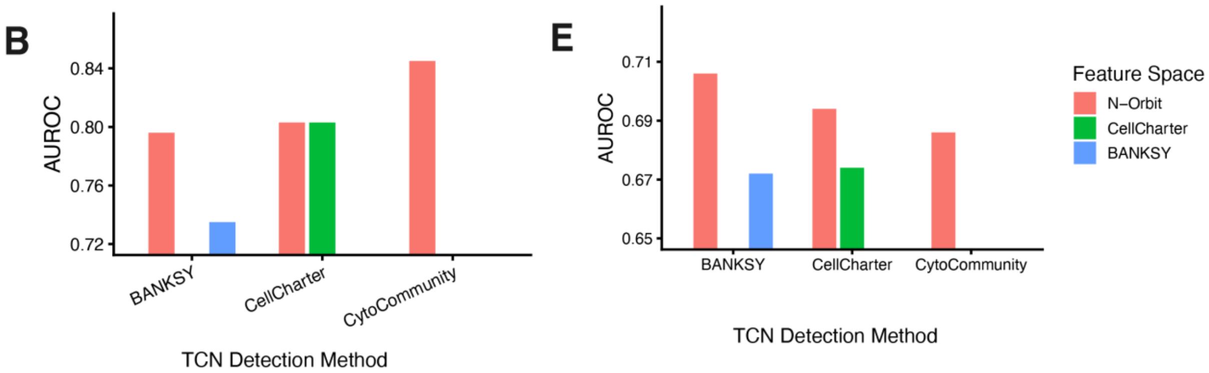


**Supplementary Figure 4. Additional benchmarking for TNBC and NSCLC datasets.** B) TNBC outcome prediction performance on alternative feature spaces. **E)** NSCLC outcome prediction performance on alternative feature spaces.

***Biological applications aspect***

6. To help readers understand and translate the N-Orbit framework into their own studies, I strongly encourage the authors to select 1~2 representative examples from Figures 4-6, draw a concise schematic plot to show how to use the tool for downstream analysis, and expand the corresponding Results text to a full paragraph that includes detailed information.

We appreciate the reviewer's suggestion and agree that a methods schematic applied to specific examples would be useful for reuse. We have added subpanels Figures 4A and 5A to more clearly illustrate the workflow for these analyses.

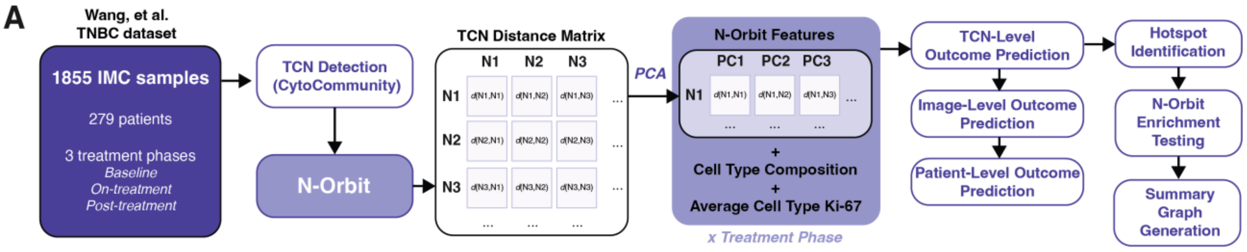


**Figure 4.** Application of N-Orbit distance to predict treatment response in triple-negative breast cancer (TNBC) patients treated with chemotherapy with or without immunotherapy. A) Methods schematic for predicting patient outcomes using N-Orbit-derived features and generating summary graphs.

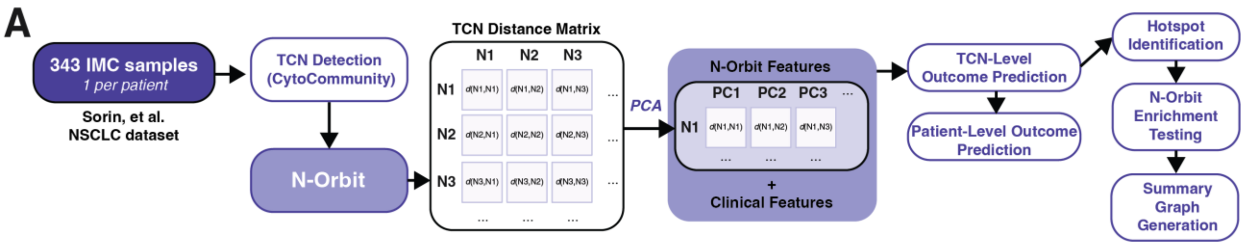


**Figure 5.** Application of N-Orbit distance to predict survival in patients with non-small cell lung cancer (NSCLC). A) Methods schematic for predicting patient outcomes using N-Orbit-derived features and generating summary graphs.

7. Take Figure 4 as an example, the following scenario needs to be presented: one TCN pair that possesses virtually identical cell-type composition (e.g., 40 % $CD8^{+}$ T, 30 % fibroblast, 30 % tumour) but occupies opposite extremes of the N-Orbit distance space (one pCR-hotspot, one RD-hotspot). Provide side-by-side zoom-in FOVs (could refer to the CytoCommunity Fig. 5d-h, https://www.nature.com/articles/s41592-023-02124-2#Fig5), their N-Orbit vector heat-maps, and the resulting pairwise distances, then conclude with a one-sentence takeaway—"same composition, different architecture, opposite clinical outcome." Such a contrast will make the added value of N-Orbit instantly intuitive to a broad readership.

We appreciate the reviewer's suggestion to provide visual examples of similar cell type compositions with varying spatial relationships and opposite outcomes. We would like to clarify that, since cell-type composition is a major factor in the structure of the N-Orbit distance space, TCN pairs with the same composition will remain relatively close in distance. TCN pairs with similar composition but different spatial relationships would likely be in a similar region of the N-Orbit space, but still further TCN pairs with both the same composition and the same spatial relationships.

We have included side-by-side examples for the TNBC and NSCLC datasets in Supplementary Figure 9.

For TNBC, the pCR example came from the pCR2 hotspot, and the RD example came from the RD2 hotspot. The two examples had a Pearson correlation of 0.86 for cell-type composition and an N-Orbit distance of 5.04. One defining feature of pCR2 is the recurrent co-membership of M2 Mac and dendritic cells that was not present in any of the RD hotspots.

For NSCLC, the NS example came from the NS1 hotspot, and the S example came from the S3 hotspot. Their cell type compositions have a Pearson correlation of 0.985 and an N-Orbit distance of 1.82. One key difference between NS1 and S3 hotspots was the recurrent co-membership of Tc and Th cells in the latter. The NS1 summary graph includes both cell types but has no edge between them, and none of the other NS summary graphs do either.

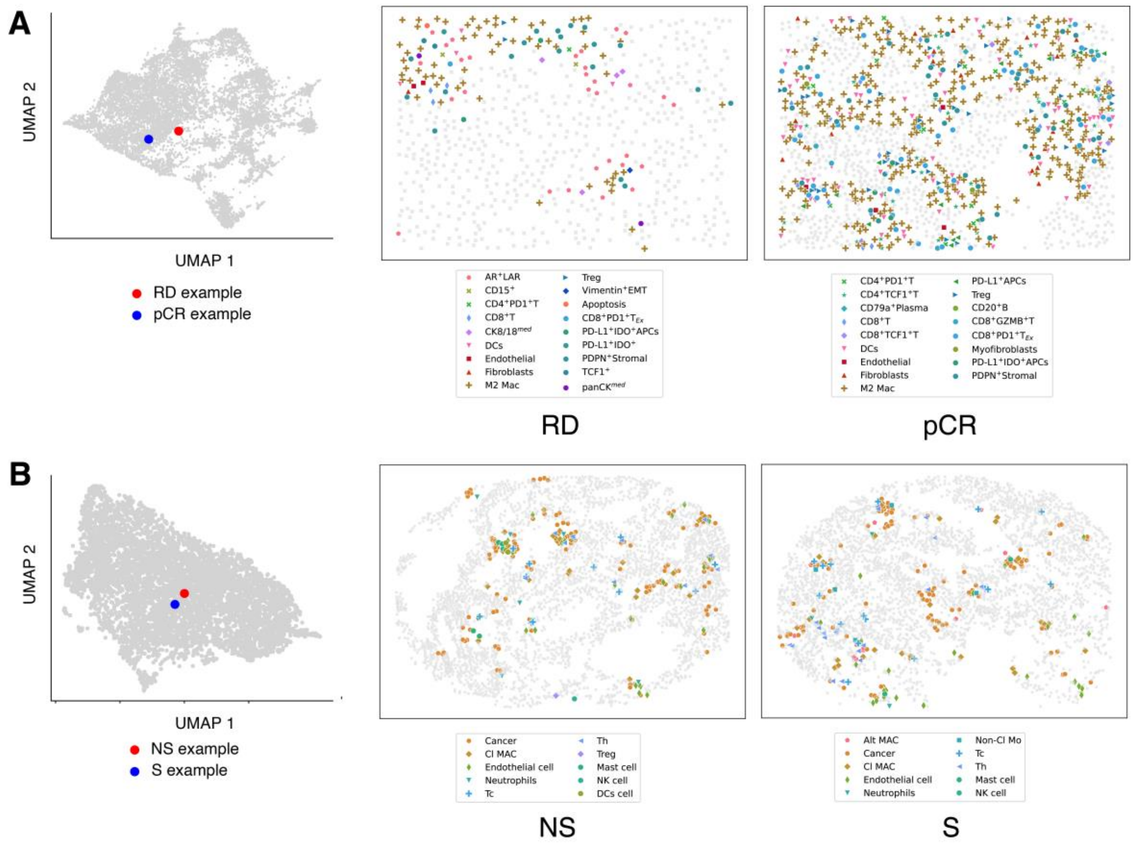


**Supplementary Figure 9: Additional examples for the TNBC and NSCLC datasets by outcome. A)** RD and pCR TCN examples with similar cell type composition (Pearson R = 0.86) but varying spatial relationships (N-Orbit distance = 5.04) from the TNBC dataset. Left, positions on the UMAP representation of N-Orbit TCN distance; Right, TCN cell type maps. Grey squares represent cells outside the TCN. **B)** NS and S TCN examples with similar cell type composition (Pearson R = 0.995) but varying spatial relationships (N-Orbit distance = 1.82) from the NSCLC dataset. Left, positions on the UMAP representation of N-Orbit TCN distance. Right, TCN cell type maps. Grey squares represent cells outside the TCN.

8. Guidance for Reuse: the concept is appealing, but the application examples in Figures 4-6 are still confusing when read in isolation and give no blueprint for reuse. I encourage the authors to further explore the mechanism of their application scenario examples and discuss what kind of scenarios are suitable for applying this method. I also suggest the authors to add a dedicated discussion subsection listing the method’s limitations

We understand the reviewer’s concern for making the methods and application digestible for the reader and facilitating reuse. We have added methods schematics in Figures 4A and 5A to provide examples for these particular datasets. Furthermore, we have added a dedicated discussion subsection regarding limitations. In addition to clarifying N-Orbit's purpose, this section clarifies the necessary inputs to run the method. It also makes note of dependence on certain upstream aspects such as quality

of cell type annotation and underlying TCNs. We have also added another discussion subsection to introduce other potential applications that were beyond the scope of this manuscript.

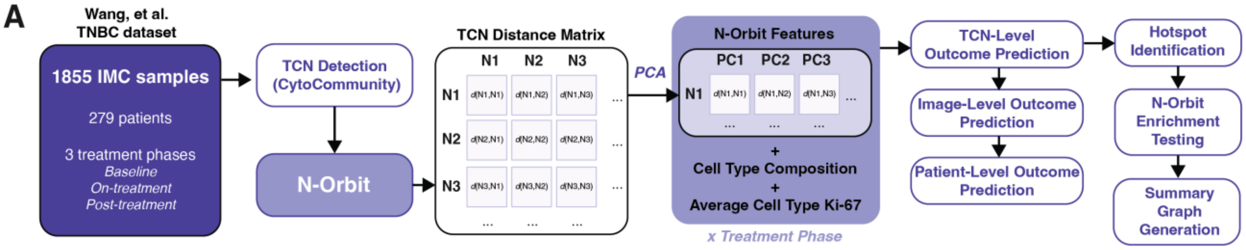


**Figure 4: Application of N-Orbit distance to predict treatment response in triple-negative breast cancer (TNBC) patients treated with chemotherapy with or without immunotherapy. A)** Methods schematic for patient outcome prediction using N-Orbit-derived features and generation of summary graphs.

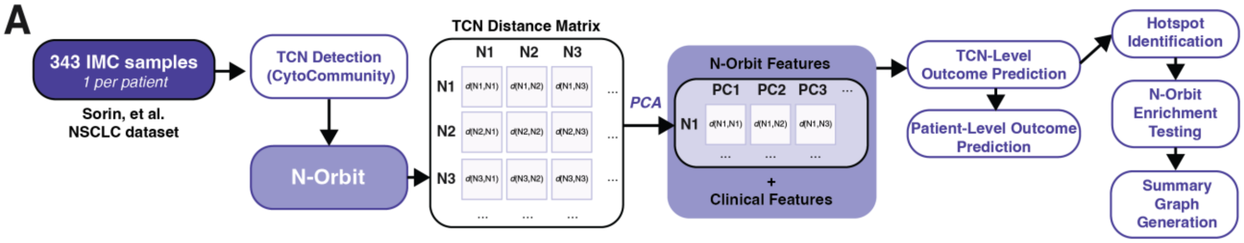


**Figure 5: Application of N-Orbit distance to predict survival in non-small cell lung cancer (NSCLC) patients. A)** Methods schematic for patient outcome prediction using N-Orbit-derived features and generation of summary graphs.

### *Minor points*

1. The term “interaction” in the manuscript does not denote a ligand–receptor pair or a spatial co-localization coefficient; instead, it is quantified by the edge weight in the N-Orbit summary graph, which simply records how frequently two cell types co-occur within the same N-Orbit. The authors should either demonstrate that this frequency metric genuinely reflects biologically meaningful cell–cell interactions or adopt a more precise term to avoid conceptual overstatement.

We understand that the term “interaction” may not be the most accurate for describing the edge weights of our summary graph. We initially intended “interaction” to denote the presence of an edge (e.g., the existence of an enriched N-Orbit with that nucleus-orbit relationship) rather than the weight itself, and “interaction strength” to denote edge weight. Nevertheless, we also recognize that the edge weight is more about the repeatability of a cell-type relationship within enriched N-Orbits than about a stronger spatial co-localization coefficient. While these may be correlated, we have revised our terminology to avoid conflating these concepts. We have replaced “interaction” with “co-membership”, referring to two cell types belonging to the same enriched N-Orbit. We have also replaced “interaction strength” with “(co-membership) recurrence”, as the

edge weights quantify the frequency of co-membership in enriched N-Orbits. We believe edge weights reflect the confidence in the relationship between two cell types as opposed to the degree of co-localization, so this terminology should reflect that more accurately.

2. As mentioned in Review #3 (regarding the concept TCN, neighborhood), the manuscript alternates between “TCNs” and “neighborhoods” without stating whether they denote the same entity. Please provide a single, explicit definition and thereafter use one term consistently; if a distinction is intended, clarify this up-front and avoid interchangeable usage.

We recognize that the interchangeable use of these two terms may be confusing. We intended them to be equivalent. TCNs are defined in the second paragraph of the introduction, and we edited to use only that term thereafter.

3. Superscripts in the manuscript and figures are typed as “^” rather than formatted text (e.g., “CD15^+”). Please replace all ASCII carets with true superscript characters throughout the main text, figure panels, and legends to meet Nature Communications production standards.

Thank you for the suggestion. They have been updated accordingly in the main text, figure panels, and legends.

Reviewer #4 (Remarks to the Author):

The authors have provided a thorough and constructive response to the initial reviewer comments. I commend the improvements made, particularly the clarification of the method's positioning within the spatial omics workflow, the revised terminology that avoids biological overinterpretation, and the inclusion of helpful schematic diagrams. These revisions significantly enhance the clarity of the manuscript. However, while the technical performance benchmarks have been strengthened, several key concerns regarding the biological interpretability and practical usability of N-Orbit remain to be fully addressed.

Technical / benchmark evaluation:

1. (For previous Q1) The authors provide a reasonable ablation test comparing N-Orbit to a density-based alternative (Supplementary Figure 3G). However, the description lacks critical methodological details that prevent full assessment of this comparison. Furthermore, the hypothalamus results in Figure S3G imply that the density-based approach occasionally outperforms N-Orbit, raising the question of whether integrating density information into N-Orbit could expand the method's applicability.

We have added text explaining the definition of the density-based distance metric and how it was used to calculate neighborhood distance. Please see the section "Calculation of density-based TCN distances" in Methods.

We acknowledge that the density-based metric performs better at smaller radii on the hypothalamus dataset. We believe this is due to two factors: first, the density-based metric can capture the density information more accurately at smaller radii but becomes less accurate at larger radii. This is also reflected in the gradual decline in performance with increasing radius. Second, the hypothalamus TCNs are generally smaller than the spleen and lung TCNs, making them better captured by the density-based metric. Additionally, this dataset contains far fewer cells than the other two tested (26K vs. 244K and 790K), with cells more sparsely distributed.

2. (For previous Q3 and Q4) While the authors have shown that N-Orbit consistently outperforms CTE across several upstream TCN callers (e.g., BANKSY, CellCharter), the rebuttal still lacks a rigorous quantitative assessment of the variance introduced by these different inputs. It is insufficient to merely state that results are 'similar' or that N-Orbit remains superior to a baseline.

To justify N-Orbit as a robust, alignment-free metric, the authors must provide a detailed benchmarking of the cross-method stability. Specifically:
(1) Please quantify the degree of divergence in N-Orbit output features (e.g., pairwise distance matrices or embedding distributions) when using different TCN callers. A simple comparison of AUROC is not enough; a more granular analysis, such as a concordance study of the resulting spatial embeddings, is required to measure how much the 'choice of caller' shifts the N-Orbit coordinate space.

We recognize the reviewer's request for a more granular demonstration of consistency in pairwise N-Orbit distances using various TCN callers. Below, we provide such results for the Spleen and Hypothalamus datasets. First, we calculated the mean distance between each pair of neighborhoods (e.g., B-zone and PALS for the spleen) and their Pearson correlations across TCN callers (Ground Truth, BANKSY, CellCharter). For TCN caller comparisons across both the Spleen and Hypothalamus datasets, correlations were very strong, ranging from 0.75 to 0.95. Secondly, we plotted histograms of pairwise distances generated by the three TCN callers. We observed a high degree of overlap for both the Spleen and Hypothalamus datasets. For the Spleen dataset, Ground Truth and CellCharter showed particularly strong overlap, and for the Hypothalamus dataset, CellCharter and BANKSY showed particularly strong overlap. Note that the adjusted mutual information (AMI) between the underlying CellCharter and BANKSY TCNs and Ground Truth TCNs was 0.58 and 0.52 on the Spleen dataset, respectively, and 0.30 vs. 0.24 on the Hypothalamus dataset, respectively.

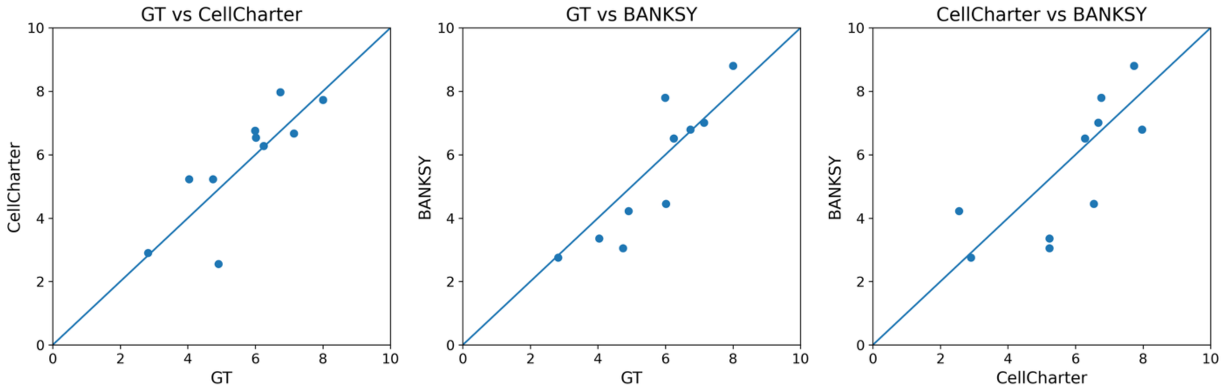


Reviewer Figure 1. Correlation of mean between-TCN distances for the Spleen dataset using ground truth (GT) vs. CellCharter vs. BANKSY for TCN calling. Pearson correlations are 0.83, 0.87, and 0.75 from left to right.

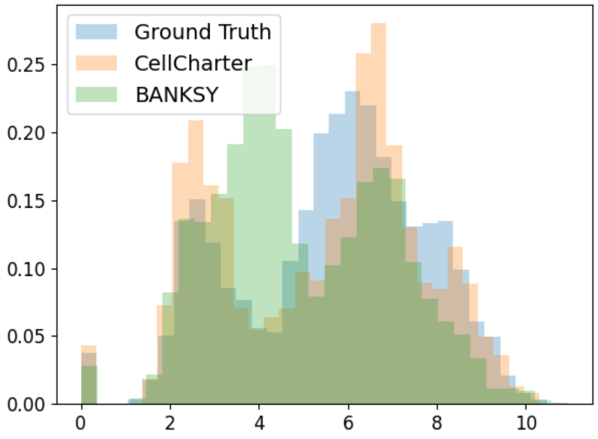


Reviewer Figure 2. Histograms of pairwise TCN distances in TCNs called from ground truth vs. CellCharter vs. BANKSY (Spleen dataset).

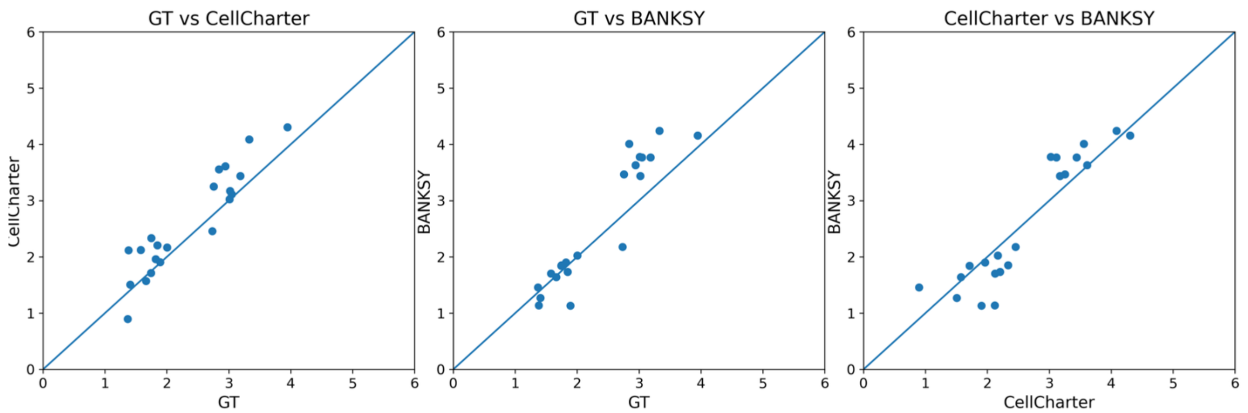


Reviewer Figure 3. Correlation of mean between-TCN distances for the Hypothalamus dataset using ground truth vs. CellCharter vs. BANKSY for TCN calling. Pearson correlations are 0.94, 0.95, and 0.93 from left to right.

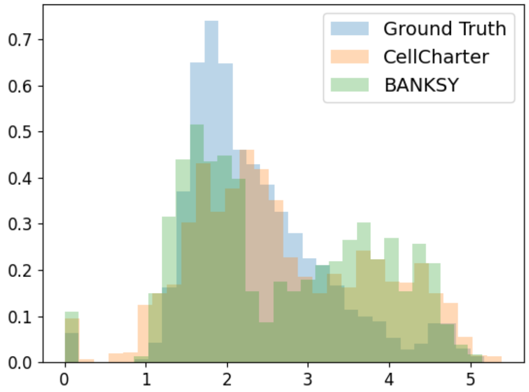


Reviewer Figure 4. Histograms of pairwise TCN distances in TCNs called from ground truth vs. CellCharter vs. BANKSY (Hypothalamus dataset).

(2) The authors should evaluate whether the performance gain of N-Orbit is sensitive to the granularity or the specific hyper-parameters of the upstream TCN detection. This will clarify if the metric's performance is truly intrinsic to N-Orbit or heavily buffered by the quality of the specific upstream tool used.

We appreciate the reviewer’s suggestion to test for sensitivity to hyperparameters from upstream TCN detection. Again, we tested this on the Spleen and Hypothalamus datasets for BANKSY and CellCharter. For both, we varied the kNN parameter. For BANKSY, we also varied the lambda (smoothing) parameter. For CellCharter, we also varied the number of layers in the graph neural network. We generally observed good stability in AUROC across hyperparameter combinations, particularly in settings that yielded concordance with the Ground Truth. N-Orbit consistently outperformed CTE across all hyperparameter settings. Regarding the general accuracy of each method for each dataset, both CellCharter and BANKSY TCNs had an AMI of 0.5-0.6 relative to Ground Truth on the Spleen dataset. We observed higher AMIs with CellCharter on the Hypothalamus dataset (~0.35) than with BANKSY (~0.2). Note that some

TCNs in the Hypothalamus dataset could not be detected by TCN callers (particularly BANKSY), which resulted in inflation of AUROC scores for certain settings.

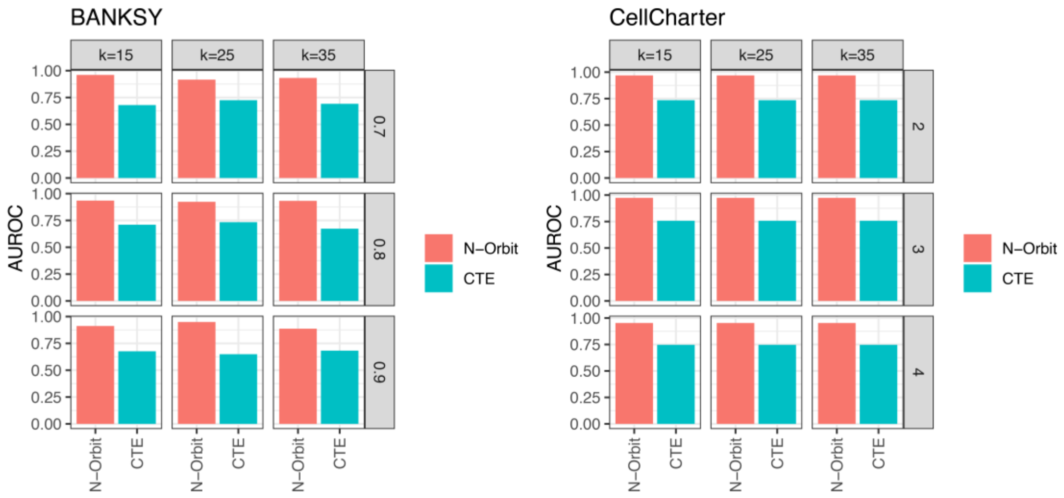


Supplementary Figure 3H. Hyperparameter testing for N-Orbit on BANKSY and CellCharter TCNs on the spleen dataset. Facet columns are kNN parameters for both BANKSY and CellCharter. Facet rows are the lambda parameter for BANKSY and the number of graph neural network layers for CellCharter.

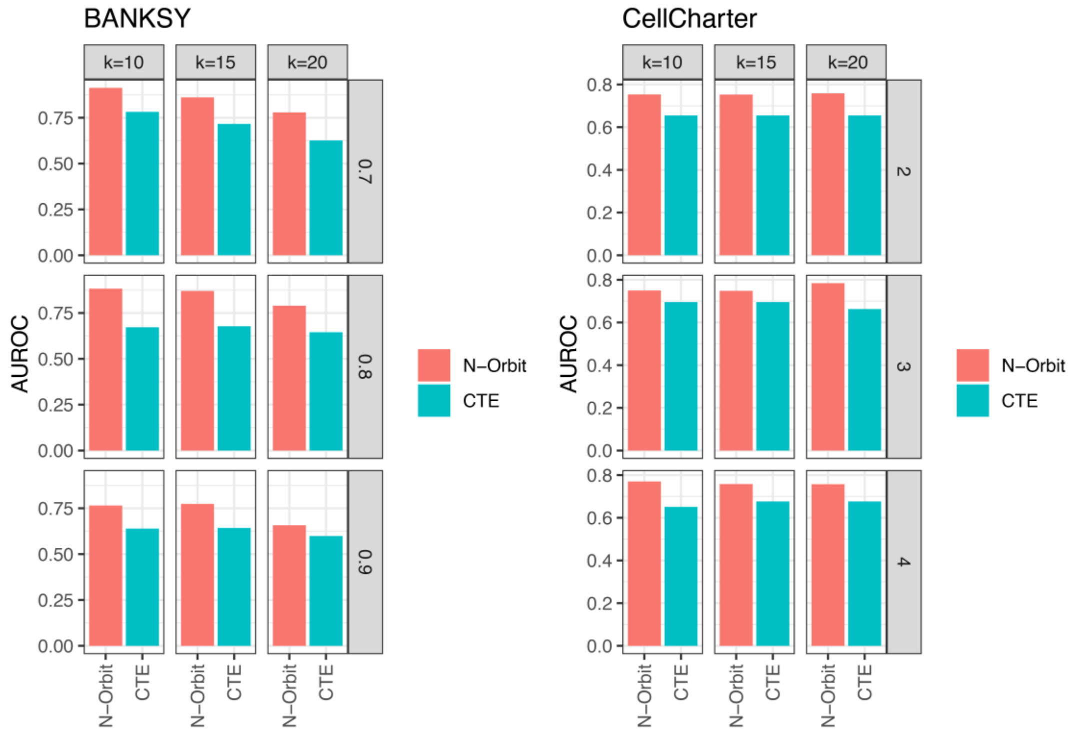


Supplementary Figure 3I. Hyperparameter testing for N-Orbit on BANKSY and CellCharter TCNs on the hypothalamus dataset. Facet columns are kNN parameters for both BANKSY and CellCharter. Facet rows are the lambda parameter for BANKSY and the number of graph neural network layers for CellCharter.

Biological applications aspect:

3. (For previous Q3, Q4, and Q8) Beyond technical benchmarks, my primary concern remains the convergence of biological discoveries. A universal metric should ideally lead to the same biological interpretation regardless of the upstream preprocessing, yet this 'universality' has not been sufficiently demonstrated.
If the downstream biological findings—such as specific cell-type co-occurrence patterns, spatial domain signatures, or patient clinical stratifications—shift significantly depending on the TCN caller, the utility of N-Orbit as a plug-and-play metric is limited.

To address this, please (1) demonstrate whether the top-ranked biological features or hotspots identified in the TNBC dataset are preserved across different TCN frameworks.

We understand the reviewer's concern about the consistency of biological interpretation across TCN callers. Below, we provide Venn diagrams of the top 10 cell type pairs (ranked by edge weight) across all 3 TCN callers (CytoCommunity, CellCharter, BANKSY) for RD and pCR TCNs in the TNBC dataset, as well as NS and S TCNs in the NSCLC dataset. Across all groups, most cell type pairs were shared by at least 2 of the 3 TCN callers, with at least half of each TCN caller's top 10 being shared among all three.

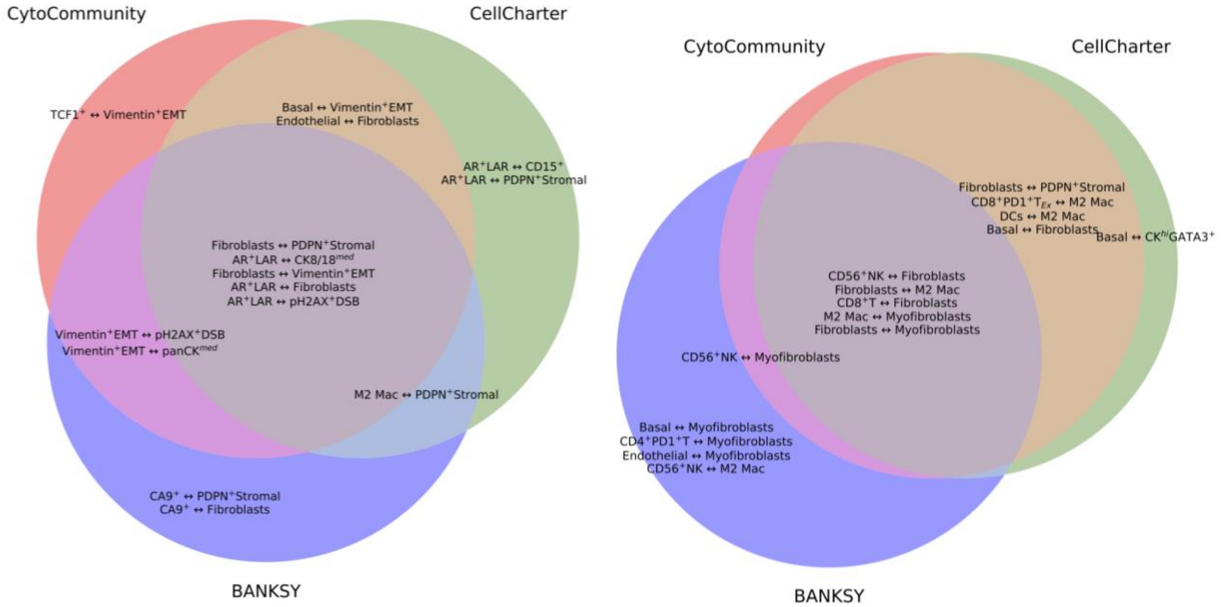


Supplementary Figure 18A-B. Venn diagram of the top 10 cell type pairs in RD (left) and pCR (right) TCNs from the TNBC dataset by TCN caller.

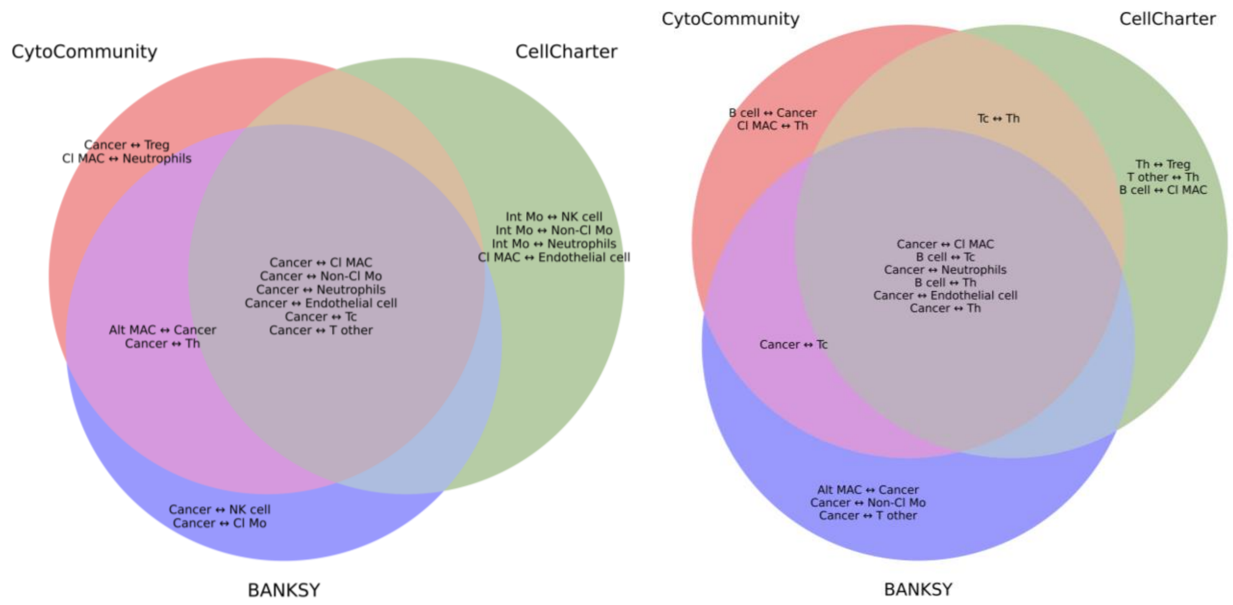

Supplementary Figure 18C-D. Venn diagram of top 10 cell type pairs in NS (left) and S (right) TCNs from the NSCLC dataset by TCN caller.

(2) In light of the observed input-dependency, the authors must provide a principled guideline for selecting upstream TCN methods. For instance, in which tissue contexts (e.g., highly structured vs. diffuse) is a graph-based caller (like CellCharter) preferred over a spatial-neighbor-based one (like BANKSY) when using N-Orbit? Why?

We acknowledge the reviewer's recommendation to provide guidelines for suggesting upstream TCN methods. This has been added to the Discussion section. Ultimately, we believe the best TCN caller is the one with the highest biological accuracy (independent of its relative compatibility with N-Orbit). Across our analysis, we found several examples of CellCharter showing better concordance with ground truth through both N-Orbit-independent (e.g., AMI) and N-Orbit-dependent (e.g., AUROC) metrics. We also observed greater overlap in biological findings between CellCharter and CytoCommunity than between CellCharter and BANKSY, particularly for the TNBC pCR TCNs. As a result, we generally recommend graph-based callers such as CellCharter and CytoCommunity for use with N-Orbit.

(3) Please include a dedicated discussion on the performance divergency from upstream callers to N-Orbit. Users need to understand the expected variance in biological output and the potential risks of unreal discoveries if a suboptimal TCN framework is chosen for a specific biological system.

We appreciate the reviewer's recommendation to emphasize the importance of the choice of upstream caller and its potential impact on results and biological findings. We draw from our findings in this revision, particularly in Points 2.2 and 3.1, to expand our discussion into the expected variance in results based on TCN caller choice.

Reviewer #4 (Remarks on code availability):

Remarks on code availability:
4. Additionally, I have tested the N-Orbit codebase available on GitHub. While the core algorithm is functional, the output matrices lack necessary row and column labels, rendering them difficult for users to interpret or integrate into downstream pipelines. Furthermore, the lack of clear documentation on output interpretation hinders the utility of the tool.
To improve reproducibility and community accessibility, I strongly recommend that the authors:
(1) Enhance code documentation: Include clear row/column headers in all output matrices and provide a detailed explanation of the output format in the documentation.

Expanded documentation has been added to the code, including descriptions of inputs/outputs for all functions. In addition, outputs of each script are now explicitly described in the README for the GitHub repo.

Intermediate outputs (e.g., Steps 1 and 2a) are not intended for user viewing and are primarily for storage between steps. Nevertheless, we added more detail in the README about what these files contain and what the column labels refer to. For example, some of these are just collections of N-Orbit vectors, with the column/row labels simply indices. They are stored as CSVs for convenience and to include metadata. The final output (neighborhood distance matrix) includes explicit row and column labels for the corresponding images and neighborhoods, enhancing interpretability.

(2) Provide a demo pipeline: Include a representative, ready-to-run sample dataset (e.g., a small subset of the TNBC or NSCLC data) with a corresponding tutorial (script or notebook). This will allow users to verify their installation and understand the expected input/output structure.

We included an example synthetic dataset (from Figure 2) that can be run in a reasonable amount of time to test proper software installation and to understand the input and output formats. The repo's file structure and the script's default settings are aligned with this dataset. We also included a Jupyter Notebook under tutorial/SyntheticV1Full.ipynb that contains command-line blocks to run the full distance pipeline (and main benchmarking analysis) fresh out of the box. For the enrichment pipeline, we also provided an example input CSV file for one cluster from the NSCLC dataset.
